# Enhancing SARS-CoV-2 Lineage Surveillance through the Integration of a Simple and Direct qPCR-Based Protocol Adaptation with Established Machine Learning Algorithms

**DOI:** 10.1021/acs.analchem.4c04492

**Published:** 2024-11-04

**Authors:** Cleber Furtado Aksenen, Debora Maria Almeida Ferreira, Pedro Miguel Carneiro Jeronimo, Thais de Oliveira Costa, Ticiane Cavalcante de Souza, Bruna Maria Nepomuceno Sousa Lino, Allysson Allan de Farias, Fabio Miyajima

**Affiliations:** † Department of Biotechnology, Oswaldo Cruz Foundation, Eusébio 61773-270, Brazil; ‡ Department of Medicine, Federal University of Ceará, Fortaleza 60430-160, Brazil; § Department of Biochemistry and Molecular Biology, Federal University of Ceará, Fortaleza 60455-760, Brazil

## Abstract

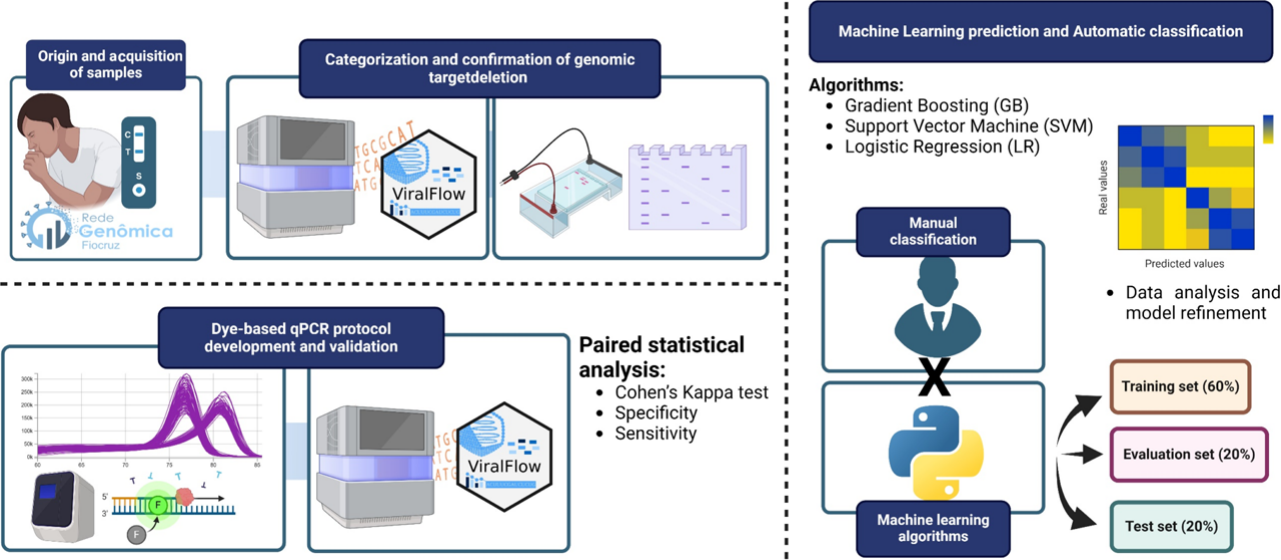

Emerging and evolving Severe Acute Respiratory Syndrome Coronavirus 2 (SARS-CoV-2) lineages, adapted to changing epidemiological conditions, present unprecedented challenges to global public health systems. Here, we introduce an adapted analytical approach that complements genomic sequencing, applying a cost-effective quantitative polymerase chain reaction (qPCR)-based assay. Viral RNA samples from SARS-CoV-2 positive cases detected by diagnostic laboratories or public health network units in Ceará, Brazil, were tracked for genomic surveillance and analyzed by using paired-end sequencing combined with integrative genomic analysis. Validation of a key structural variation was conducted with gel electrophoresis for the presence of a specific *open reading frame 7a*(*ORF7a*) gene deletion within the “BE.9” lineages tracked. The analytical innovation of our method is the optimization of a simple intercalating dye-based qPCR assay through repositioning primers from the ARTIC v4.1 amplicon panel to detect large molecular patterns. This assay distinguishes between “BE.9” and “non-BE.9” lineages, particularly BQ.1, without the need for expensive probes or sequencing. The protocol was validated against lineage predictions from next-generation sequencing (NGS) using 525 paired samples, achieving 93.3% sensitivity, 95.1% specificity, and 92.4% agreement, as measured by Cohen’s Kappa coefficient. Machine learning (ML) models were trained using the melting curves from intercalating dye-based qPCR of 1724 samples, enabling highly accurate lineage assignment. Among them, the support vector machine (SVM) model had the best performance and after fine-tuning showed ∼96.52% (333/345) accuracy in comparison to the test data set. Our integrated approach provides an adapted analytical method that is both cost-effective and scalable, suitable for rapid assessment of emerging variants, especially in resource-limited settings. In this work, the protocol is applied to improve the monitoring of SARS-CoV-2 sublineages but can be extended to track any key molecular signature, including large insertions and deletions (indels) commonly observed in pathogenic agent subtypes. By offering a complement to traditional sequencing methods and utilizing easily trainable machine learning algorithms, our methodology contributes to enhanced molecular surveillance strategies and supports global efforts in pandemic control.

## Introduction

The rapid adaptation of viruses through cumulative mutations highlights the dynamic interactions between pathogens and their environments. Structural modifications resulting from insertions or deletions can lead to significant changes in viral behavior, ultimately fueling the emergence of variants with potential selective advantages and altered pathogenic profiles.^1−3^ This adaptive mechanism has been illustrated by the emergence of SARS-CoV-2 variants, which are known for their increased infectivity due to specific amino acid modifications.^4,5^ The genetic diversity observed in RNA viruses, underscored by the continuous emergence of new mutations, highlights the evolving nature of these pathogens and the critical role of genomic surveillance in tracking these changes.^3,6,7^

The swift emergence and global proliferation of the Omicron variant (B.1.1.529) and its sublineages have raised public health concerns due to their unique genetic configurations and unprecedented transmission rates.^8,9^ Studies have shown that Omicron can outcompete previous strains such as the Delta variant, in terms of spread, leading to increased reinfection rates, affecting even among vaccinated or previously infected individuals.^10−13^ The variant’s severity profile compared with its predecessors necessitates rigorous public health interventions.^14−16^ Given the dynamic landscape, there is an imperative need for genomic surveillance to track and understand the emergence of new strains, enabling proactive measures to mitigate their impact.

Global efforts to monitor and control the spread of SARS-CoV-2 face numerous challenges, including economic and infrastructural disparities among countries. Next-generation sequencing (NGS), while invaluable for detailed genomics analysis, is often limited by high costs, lengthy turnaround times, and limited accessibility in resource-constrained regions. These limitations have prompted the scientific community to explore supplementary analytical techniques.^17−19^ There has been a shift toward integrating quantitative polymerase chain reaction (qPCR) and computational algorithms into the genomic surveillance framework. These methods offer a more immediate and cost-effective capability for detecting specific genetic markers, thereby enhancing the efficiency and reach of pathogen monitoring efforts.^20,21^

Amidst the array of innovative techniques in genetic surveillance, the use of an intercalating dye-based qPCR protocol has emerged as an important analytical adaptation. Distinguished by its capacity for real-time DNA amplification monitoring and its low cost, this protocol has demonstrated remarkable efficacy in identifying specific genetic markers. Its application not only represents a strategic advancement in the rapid identification of variants of concern (VOCs) but also enhances our understanding of the intricate dynamics of viral adaptations.^22,23^ Specifically, insights gained into the *ORF7a* gene, known for its role in immune modulation and host cell interaction, underscore the complex interplay between viral genetics and host defenses. This emphasizes the importance of nuanced genetic surveillance in preparing for challenges posed by the pandemic and future viral threats.^3,24,25^

Our study introduces an adapted analytical method utilizing an intercalating dye-based qPCR protocol as a low-cost assay, adaptable for near-real-time genomic surveillance. This strategy can significantly improve both the speed and precision for target detections, proving reliable for confirming key molecular signatures used in tracking the population dynamics and evolution of pathogens, like SARS-CoV-2. Our work has highlighted the applicability of a lineage-defining genetic marker—a 244-base deletion within the *ORF7a* gene (27,508–27,751)—characteristic of the Brazilian BE.9 lineage. By tracking this specific deletion from September 2022 to May 2023 (https://gisaid.org/), we reveal the utility of a qPCR-based protocol in monitoring the expansion of emerging variants and sublineages that pose new challenges to public health and vaccine efficacy. This seamless integration of computational analyses and a straightforward intercalating dye-based qPCR protocol represents a more direct and inclusive approach to monitoring viral evolution. This methodology gives the scientific community and public health policy makers the power to engage in rapid response measures against evolving pathogen variants, ensuring that public health strategies remain robust and responsive in combating immune escape and SARS-CoV-2 adaptability.

## Experimental Section

### Origin and Acquisition of Samples

The viral RNA samples were acquired through a collaborative initiative focused on genomic monitoring of SARS-CoV-2, conducted by the Oswaldo Cruz Foundation (Fiocruz) Genomic Surveillance Network, a leading research institution under the Brazilian Ministry of Health. These samples were obtained from the repurposing of rapid antigen tests conducted as part of routine clinical care, screening processes, and active surveillance for variants in hospitals and health centers in the state of Ceará, Brazil.

### NGS and Lineage Classification

NGS was performed as part of the routine genomic surveillance of SARS-CoV-2. Paired-end sequencing libraries were prepared using the ARTIC v4.1 primer set (https://github.com/artic-network/artic-ncov2019/blob/master/primer_schemes/nCoV-2019/V4/SARS-CoV-2.primer.bed) and the CovidSeq protocol, following the manufacturer’s recommendations. Sequencing was carried out on an Illumina NextSeq 2000 platform for all samples. The raw sequencing data were analyzed using the ViralFlow v1.0.0 workflow,^100^ which includes quality control, preprocessing, alignment of high-quality reads to the reference genome and genome assembly. Lineage classification was performed using Pangolin v4.3.1^36^ and Nextclade v3.0.1^37^ software, facilitating the identification and annotation of genetic variations.

### Integrative Genomic Analysis and Deletion Identification

To confirm the presence of the anticipated 244-base pair (bp) deletion in *ORF7a* gene, 16 high-quality sequencing samples were carefully selected based on stringent criteria: horizontal coverage exceeding 90% and average vertical coverage surpassing 100×. These samples were divided into two distinct groups: the “BE.9” group (samples S01 to S08), and the “non-BE.9” group (samples S09 to S16). Alignment files were evaluated using the Geneious Prime software, and the coverage variation throughout the genome was used to predict the deletion present in the *ORF7a* gene of BE.9 group.

The deletion was further validated through 2% agarose gel electrophoresis, performed at 90 V for 4 h to ensure optimal separation and visualization of DNA fragments. The resulting bands, including those corresponding to the targeted *ORF7a* deletion, were visualized using Thermo Fisher iBright equipment, allowing for immediate image capture and analysis.

### Intercalating Dye-Based qPCR Amplification Protocol

To categorize the samples by qPCR protocol, specific primers from the ARTIC v4.1 set were repurposed to amplify the region of interest (nucleotides 37,508–37,751) in the *ORF7a* gene Av4_SARS-CoV-2_92_LEFT (5′-CACTACCAAGAGTGTGTTAGAGGTAC-3′) and Av4_SARS-CoV-2_92_RIGHT (5′-GTTCAAGTGAGAACCAAAAGATAATAAGC-3′). This novel approach repurposes existing primers to develop a cost-effective qPCR assay capable of distinguishing between SARS-CoV-2 lineages based on a specific deletion in the *ORF7a* gene. By utilizing intercalating dye-based detection and melting curve analysis, the method circumvents the need for expensive probes or sequencing, making it particularly suitable for resource-limited settings.

An assay was conducted with the GoTaq 1-Step RT-qPCR System kit and BRYT Green Dye (Promega). The protocol was adjusted and optimized to achieve a final primer concentration of 300 nM. Each 10 μL reaction included 5 μL of Master Mix, 0.2 μL of GoScript, 0.5 μL of each primer, and 3 μL of RNA sample, optimizing reagent and sample usage in resource-constrained settings. The amplification protocol comprised an initial reverse transcription step, followed by 45 cycles of denaturation at 95 °C for 15 s, annealing at 65 °C for 30 s, and an extension step at 72 °C for 50 s. For the melting curve analysis, a high-resolution dissociation at 95 °C, with a ramp rate of 0.075 °C per second for 1 s was conducted (https://assets.thermofisher.com/TFS-Assets/LSG/manuals/MAN0013511_PowerUp_mastermix_UG.pdf). The qPCR and melting curve acquisition were performed using QuantStudio 7 Pro qPCR Systems (Thermo Fisher Scientific, Inc.). BRYT Green fluorescence was monitored during each extension cycle and in the final dissociation step, enabling real-time fluorescence measurements.

Samples were manually analyzed from Design & Analysis 2.7.0 software and categorized as “BE.9”, “non-BE.9”, or “inconclusive” based on predefined standards. Curves with melting temperatures (*T*_m_) between 76 and 78 °C and fluorescence levels above 100 units were classified as “BE.9”. Curves with *T*_m_ between 80 and 82 °C and fluorescence levels above 100 units were classified as “non-BE.9” and curves with more than two *T*_m_ peaks or fluorescence levels below 100 units were classified as “inconclusive”.

### Statistical Analysis and Protocol Validation

A total of 525 samples were included in this validation phase, where the results obtained from the intercalating dye-based qPCR protocol were compared to the conclusive lineage sequencing results. These samples were analyzed simultaneously, allowing for a direct comparison of the methods. The results were organized into a concordance table, and statistical analyses were performed using Cohen’s Kappa test to assess the agreement between the two methods, focusing on specificity and sensitivity.

### ML Algorithms and Data Analysis

A total of 1724 qPCR melting curves were manually analyzed and categorized based on the predefined standards as “BE.9”, “non-BE.9”, or “Inconclusive”. The data points from these curves were used as input for ML models training. The data set was structured into a feature matrix *X*, containing all data points from all curves, and a target vector y, containing the corresponding classification labels for each curve. The 192nd point of each curve (last column of matrix *X*) was removed because 475 samples had missing values at this position. The data were then split into training (60%, *n* = 1034), evaluate (20%, *n* = 345), and test (20%, *n* = 345) sets. Subsequently, the *X* values were normalized to a range of 0 to 1, applied for unbiased training of the models. The training data were also balanced to prevent a class imbalance. Three ML algorithms were employed for data modeling: Gradient boosting (GB), support vector machine learning (SVM), and logistic regression (LR). Models were run with default parameters, except for the SVM, where the “kernel” parameter was set to “linear” instead of default “rbf” (radial basis function). The analysis was conducted using Python 3.10.12 in conjunction with the Scikit-learn 1.4.0 library (for SVM and LR) and the XGBoost 2.0.3 package (for GB), all implemented within the Google Colaboratory environment. The code used for training the ML models is available with all steps documented in Supporting Information 1.

The model exhibiting the highest accuracy, reflecting overall correctness, underwent a grid search optimization step to fine-tune its parameters with a particular focus on optimizing accuracy. Supporting Table 1 presents the comprehensive results of the grid search, including all parameter values, along with corresponding precision, recall, F1-score, and accuracy metrics.^38^

## Results and Discussion

### Integrative Genomic Analysis and Deletion Identification

**Figure 1:**
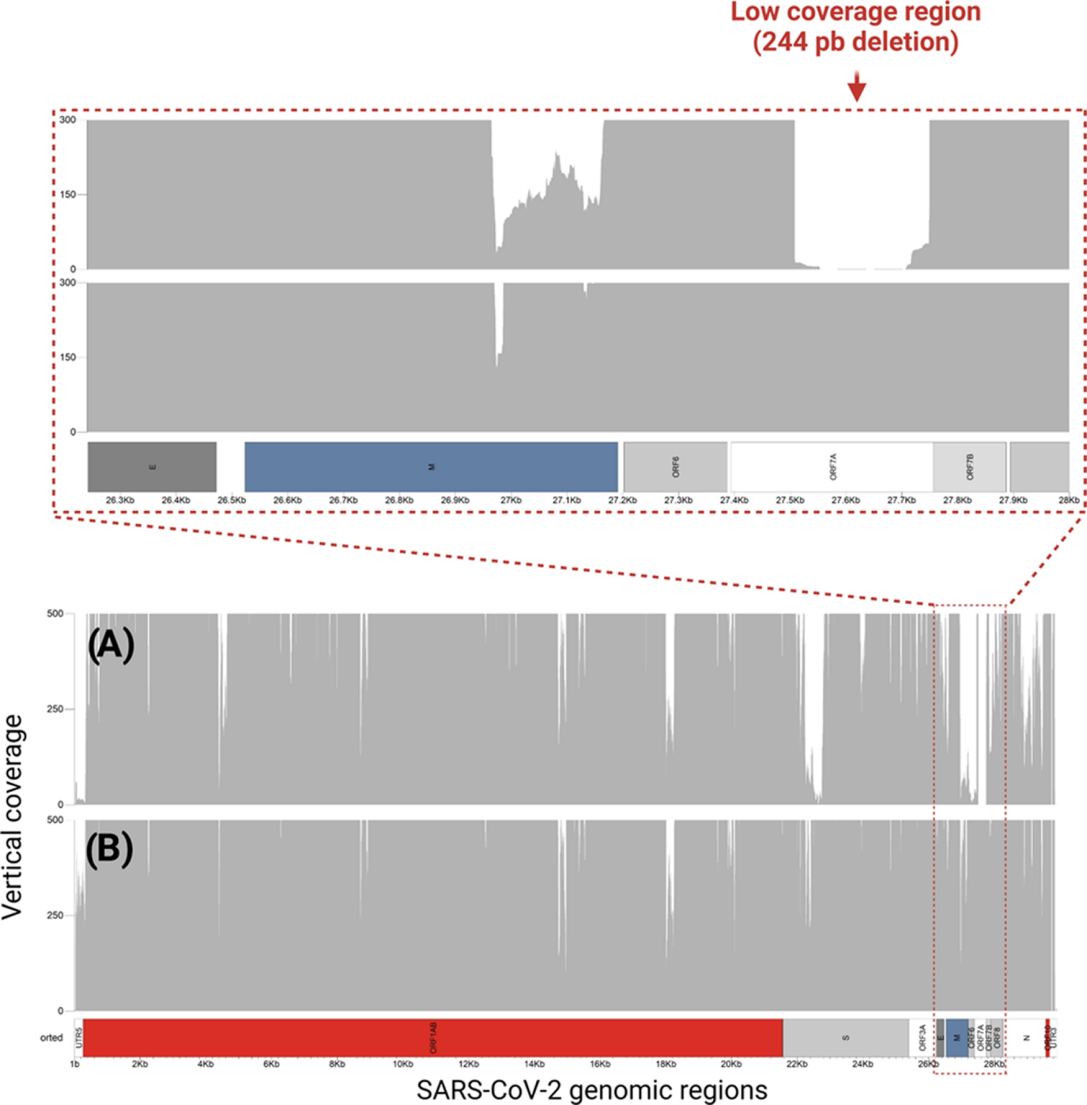
Genomic coverage profiles highlighting the *ORF7a* deletion in SARS-CoV-2 BE.9 lineages. (A, B) Sequencing coverage across the SARS-CoV-2 genome for BE.9 and non-BE.9 lineages, respectively. In BE.9 samples (A), a pronounced drop in coverage is observed in the *ORF7a* gene region (nucleotides 27,508–27,751), corresponding to the characteristic 244 bp deletion. Non-BE.9 samples (B) show consistent coverage across this region, indicating the absence of deletion.

High-quality SARS-CoV-2 genomic sequences revealed a low-depth region at positions 27,508 to 27,751 within the *ORF7a* gene in samples previously classified as “BE.9” (Figure 1A), compared to samples classified as “non-BE.9”, particularly the BQ.1 lineage (Figure 1B). The presence of extensive low-depth regions in sequencing data poses challenges to bioinformatic analysis and interpretation, undermining the accurate identification of evolutionary events, such as deletions.

**Figure 2:**
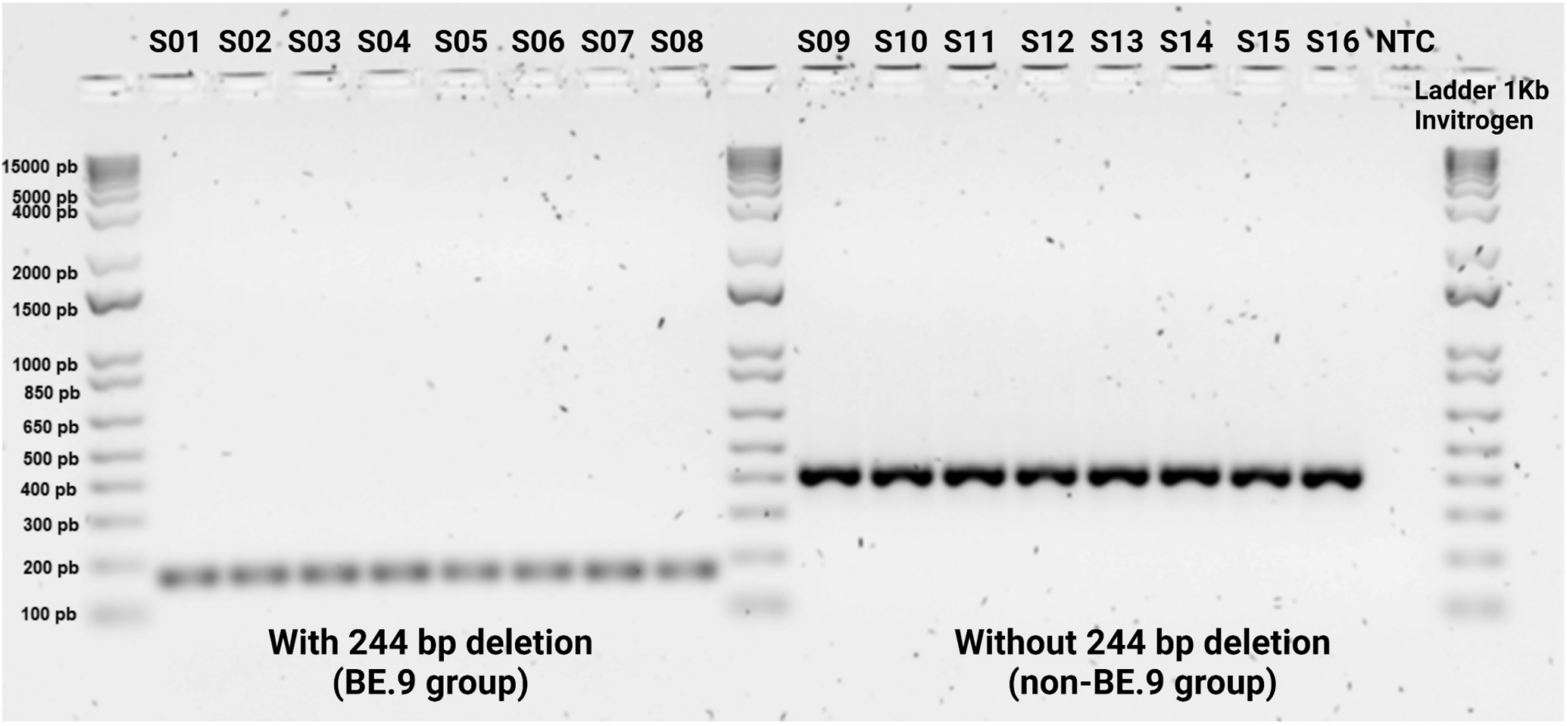
Agarose gel electrophoresis validating the ORF7a deletion in BE-9 samples. Electrophoresis results of amplified DNA fragments from SARS-CoV-2 samples. Lanes S01 to S08 represent BE.9 lineage samples, showing smaller DNA fragments around 170–200 bp due to the 244 bp deletion in the *ORF7a* gene. Lanes S09 to S16 represent non-BE.9 samples, displaying larger DNA fragments between 400 and 430 bp, corresponding to the intact *ORF7a* gene. The last lane shows the negative control (NTC). This size difference confirms the presence of the deletion in BE.9 samples and its absence in non-BE.9 samples.

To corroborate the detection of the structural mutation in the *ORF7a* gene, gel electrophoresis on the amplified targets confirmed the deletion as a synapomorphic signature of the BE.9 lineage. Specifically, a characteristic band in the range of 170–200 base pairs (bp) was consistently observed across all BE.9 samples (S01 to S08) that were phylogenetically assigned by whole-genome sequencing. In contrast, among the “non-BE.9” samples (S09 to S16), bands corresponding to *ORF7a* fragments between 400 and 430 bp were consistently present, indicating the absence of the deletion (Figure 2).

These distinct band patterns provide compelling evidence of genuine structural alterations between two major SARS-CoV-2 sublineages of independent origins. This reinforces findings from previous studies concerning the loss of genetic elements during the natural evolution of SARS-CoV-2^3^. Additionally, this approach demonstrates the applicability of a PCR amplification protocol employing intercalating dye-based strategies, which enhances the speed and robustness of variant detection efforts.

### Intercalating Dye-Based qPCR Amplification Protocol

The qPCR adapted analytical method provides a targeted and high-throughput approach for distinguishing between “BE.9” and “non-BE.9” lineages. It is reliable, flexible, and cost-effective, making it suitable for widespread genomic surveillance.^26,27^

**Figure 3:**
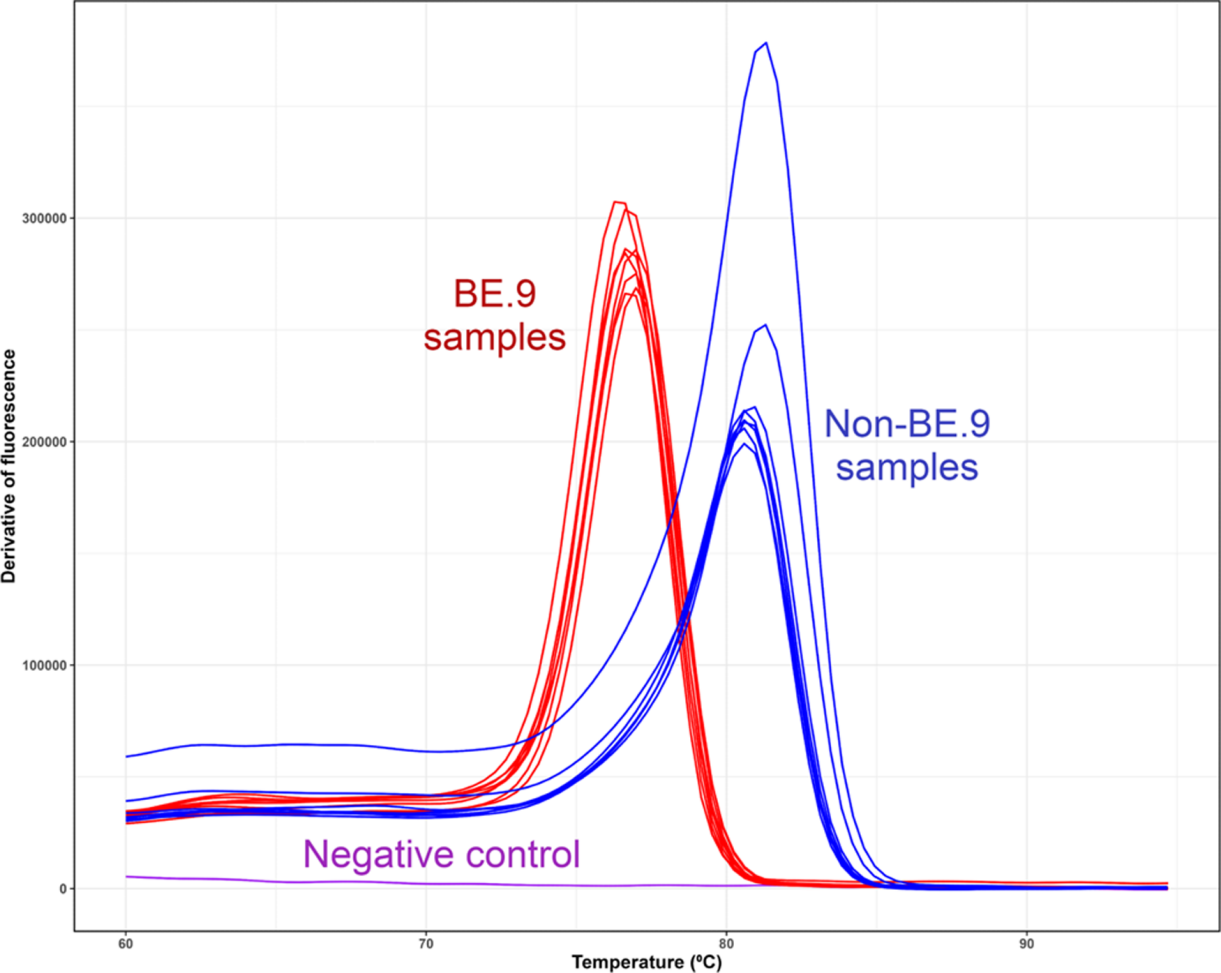
Melting curve analysis for detection of the *ORF7a* 244 bp deletion via intercalating dye-based qPCR. The figure shows the first derivative melting curves from the qPCR assay targeting the 244 bp deletion of the *ORF7a* gene. BE.9 samples (red curves) consistently exhibit lower *T*_m_ of 76.78  ±  0.18 °C due to the shorter amplicon size resulting from the deletion. Non-BE.9 samples (blue curves) display higher *T*_m_ values of 80.76  ±  0.24 °C, corresponding to the longer intact amplicon. The negative control (purple curve) shows no amplification, confirming the assay’s specificity.

The results from the first derivative melt curves of the 16 samples are shown in Figure 3, illustrating the melt curves corresponding to the BE.9 group and the non-BE.9 group. All melt curves for the BE.9 group exhibited amplification at an average melting temperature (*T*_m_) of 76.78 ± 0.18 °C, with fluorescence levels ranging between 200,000 and 300,000 units at the peak. In contrast, the non-BE.9 group displayed an average *T*_m_ of 80.76 ± 0.24 °C, with fluorescence levels spanning from 200,000 to 400,000 units. This distinction, highlighted by the lower *T*_m_ for BE.9 and higher *T*_m_ for non-BE.9, aligns with prior research suggesting that longer amplicons exhibit higher *T*_m_ compared to shorter ones.^28,29^ Notably, during manual analysis and categorization of the samples, melting curves with fluorescence levels below 100,000 were challenging to visualize and classify accurately. This challenge was significantly alleviated when we focused on the fluorescence levels above 100,000 units, enhancing the clarity and precision of group classification.

The first derivative melt curves demonstrated a distinct separation between the BE.9 and non-BE.9 groups with no overlap in *T*_m_ ranges between groups. This underscores the assay’s effectiveness in distinguishing between viral lineages. The negative control (NTC) visualized in Figure 3, which represents the absence of the virus, exhibited no fluorescence, indicating the absence of primer dimers or unintended amplification products in the assay. This highlights the assay’s reliability and the careful management of primers, minimizing the presence of contaminating artifacts.

### Statistical Analysis and Protocol Validation

The optimized protocol demonstrated significant efficiency in lineage classification compared to NGS data, as evidenced by the Cohen’s Kappa coefficient (κ = 0.924, *p*-value < 0.0001). This high level of agreement indicates that the qPCR protocol is reliable and can effectively replicate the lineage classification results obtained from NGS. The sensitivity of the protocol was 93.3%, indicating a high true positive rate for identifying BE.9 lineages. The specificity was 95.5%, reflecting a low false positive rate in detecting non-BE.9 lineages (Table 1). These values demonstrate that the qPCR assay is highly effective in accurately identifying both positive and negative cases, offering a rapid and cost-effective alternative to NGS for lineage classification.

**Table 1:** Concordance Matrix Comparing qPCR Protocol Classification with NGS Lineage Predictions for the BE.9 and Non-BE.9 Samples^a^

	NGS lineage prediction	
qPCR protocol	BE.9	non-BE.9
BE.9	169	15
non-BE.9	5	296
inconclusive	12	28

a This table presents the agreement and discrepancies between the qPCR assay results and NGS lineage assignments for BE.9 and non-BE.9 samples.

### ML Algorithms and Data Analysis

The SVM with a linear kernel emerged as the best-performing model, surpassing the other algorithms in classification accuracy (Table 2).

**Table 2:** Performance Metrics of ML Algorithms for qPCR Melting Curve Classification^a^

	precision			recall			F1-score			
algorithm	BE.9	non-BE.9	inconclusive	BE.9	non-BE.9	inconclusive	BE.9	non-BE.9	inconclusive	accuracy
SVM	0.991	0.992	0.936	0.974	0.968	0.981	0.983	0.980	0.960	0.974
logistic regression	1.000	0.976	0.910	0.932	0.984	0.971	0.965	0.980	0.940	0.963
gradient boosting	0.983	0.976	0.961	0.983	0.984	0.952	0.983	0.980	0.957	0.974

a The table compares the performance of three machine learning algorithms, SVM, GB, and LR, in classifying qPCR melting curve data into “BE.9”, “non-BE.9”, and “Inconclusive” groups. Metrics include accuracy, precision, recall, and F1-score for each algorithm, highlighting the superior performance of the SVM model.

Optimizing the SVM parameters resulted in a tie between various hyperparameter configurations (Supporting Table 1). Table 3 compares the unoptimized version of the SVM model with the one using the settings {“C”: 100, “degree”: 2, “kernel”: rbf, γ: auto} on the evaluate set with the fine-tuned model on the test set. The accuracy demonstrated a marginal improvement, accompanied by enhanced precision, recall, and F1-score metrics for certain subsets within the BE.9, non-BE.9, or inconclusive groups when evaluating the impact of fine-tuning on the test set. This uptick in accuracy indicates the accurate classification of previously mislabeled inconclusive curves as non-BE.9. However, it is imperative to assess these metrics using unseen data. While there was a decrease in metrics, it is probable that these values reflect the true performance on other unseen data sets.

**Table 3:** Performance Comparison of the SVM Model before and after Hyperparameter Optimization^a^

	precision			recall			F1-score			
algorithm	BE.9	non-BE.9	inconclusive	BE.9	non-BE.9	inconclusive	BE.9	non-BE.9	inconclusive	accuracy
SVM before tuning (evaluate set)	0.991	0.992	0.936	0.974	0.968	0.981	0.983	0.980	0.958	0.974
SVM after tuning (evaluate set)	1.000	0.969	0.980	0.992	0.992	0.962	0.996	0.980	0.971	0.983
SVM after tuning (test set)	0.989	0.957	0.955	0.949	1.000	0.939	0.968	0.978	0.947	0.965

a This table presents the performance metrics of the SVM model before and after hyperparameter optimization. The unoptimized model used default parameters, while the optimized model employed specific settings: penalty parameter C = 100, degree = 3, and kernel function set to “linear”. Metrics include accuracy, precision, recall, and F1-score for the classification of “BE.9”, “non-BE.9”, and “Inconclusive” groups based on qPCR melting curve data.

**Figure 4:**
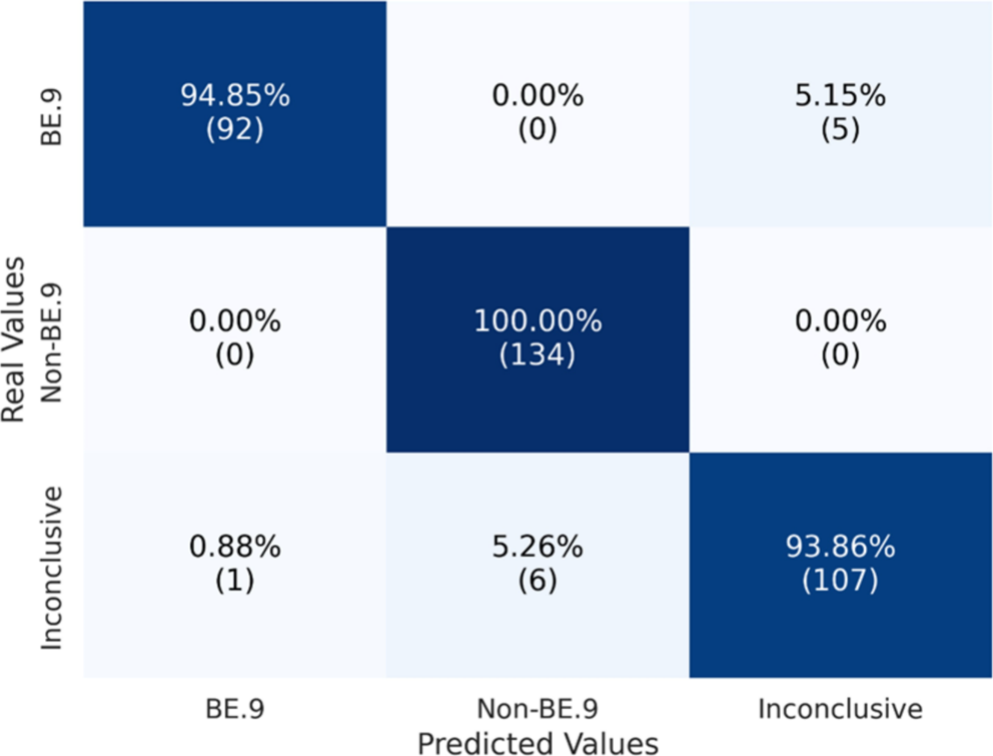
Confusion matrix illustrating the performance of the optimized SVM model on melting curve classification. The figure displays the confusion matrix for the optimized SVM model applied to the qPCR melting curve data. The model demonstrates high classification accuracy for “BE.9”, “non-BE.9”, and “Inconclusive” samples, indicating substantial reliability in automating the classification process using machine learning.

The high accuracy signifies substantial reliability when utilizing melting curve points for curve classification, automating the process. Other studies have approached diagnostic classification using derived metrics, whether through principal component analysis (PCA),^30,31^ metrics related to curve shape (skewness, kurtosis, etc.),^20,32^ our approach directly utilizes the raw data points from the curves. This simplifies the model development process while maintaining high accuracy. The clear distinction between the melting curves for BE.9 and non-BE.9 lineages enables this direct approach, allowing the model to discern features for accurate classification. The confusion matrix reveals correct classification values for the BE.9 lineage at 94.85% (*n* = 92), non-BE.9 at 100.00% (*n* = 134), and inconclusive at 93.86% (*n* = 107) (Figure 4). Despite the high classification accuracy for SARS-CoV-2 BE.9 and non-BE.9 lineages, there is a noticeable decline in the classification quality for inconclusive curves, often reflecting the subjective nature of classification by analysts. It is crucial, therefore, during the establishment of the gold standard used for model training to clearly define each of the curves.

The use of accessible platforms such as Google Colaboratory for model development and deployment contributes to the democratizing and accelerating outbreaks investigations in regions lacking computational resources.^33^ By utilization of minimally processed data and straightforward modeling techniques, our approach offers an opportunity for other laboratories to optimize protocols and integrate machine learning algorithms into routine laboratory procedures. This demystifies the use of machine learning in biological applications, as already demonstrated in other domains.^34,35^

## Conclusions

Our study presents a novel analytical methodology that combines an optimized intercalating dye-based qPCR protocol with ML analysis to effectively discriminate and classify closely related SARS-CoV-2 sublineages. This cost-effective approach provides automated differentiation between circulating sublineages (BE.9 or non-BE.9), serving as a valuable complement to more complex and resource-intensive NGS surveillance methods. By identifying a specific 244 bp deletion in the *ORF7a* gene of BE.9 samples, confirmed through gel electrophoresis, we demonstrated the utility of targeting structural genomic alterations for monitoring the emergence and spread of SARS-CoV-2 variants. The *T*_m_ curves between “BE.9” and “non-BE.9” groups, along with a classification sensibility of 94.85 and 100.00%, respectively, using the SVM algorithm, highlight the robustness of our methodology. Despite initial challenges with “inconclusive” samples, primarily due to the quality of reused rapid antigen test samples, our method maintained a high classification accuracy of 93.86% for these cases. These results underscore the potential of qPCR-based protocols for investigating evolutionary patterns in pathogens with broad implications for diagnostics, surveillance, and public health interventions.

Further research should focus on validating and refining our method, extending its applicability to other infectious diseases, and addressing any existing limitations. The integration of ML methodology increases the analytical capabilities of qPCR data, optimizing lineage classification and offering a scalable solution for molecular surveillance.

Exploring the potential application of nonspecific intercalating dye assays, combined with ML analysis, for detecting and identifying various pathogens opens avenues for expanding this innovative analytical methodology. This broader application not only enhances its utility but also reduces costs and the need for robust equipment, making advanced molecular surveillance more accessible to diverse research settings. Overall, our study advances analytical methodologies in infectious disease research and underscores the potential of interdisciplinary approaches to combat emerging pathogens.

## Supporting Information Available

Jupyter Notebook with the codes for training machine learning models and ranking of hyperparameters of SVM model (PDF)
