# Supplementary material for: Enhancing SARS-CoV-2 Lineage Surveillance through the Integration of a Simple and Direct qPCR-Based Protocol Adaptation with Established Machine Learning Algorithms
Source: Anal Chem. 2024 Nov 4;96(46):18537–44. doi: 10.1021/acs.analchem.4c04492 (PMC11579975; doi:10.1021/acs.analchem.4c04492)
Supplement: Supplementary file 1 — ac4c04492_si_001.pdf [file ac4c04492_si_001.pdf]

Supporting Information

Enhancing SARS-CoV-2 Lineage surveillance through the integration of a simple and direct qPCR-based protocol adaptation with established machine learning algorithms

Cleber Furtado Aksenen*[1.2#]. Debora Maria Almeida Ferreira[1.3#]. Pedro Miguel Carneiro Jeronimo[1.2#]. Thais de Oliveira Costa[1.2]. Ticiane Cavalcante de Souza[1]. Bruna Maria Nepomuceno Sousa Lino[1.2]. Allysson Allan de Farias[1.2]. Fabio Miyajima[1.2]

1 Department of Biotechnology. Oswaldo Cruz Foundation. Eusébio 61773-270. Brazil;
2 Department of Medicine. Federal University of Ceará. Fortaleza 60430-160. Brazil;
3 Department of Biochemistry and Molecular Biology. Federal University of Ceará. Fortaleza 60455-760. Brazil;
*Corresponding Author. Email: cleber.aksenen@fiocruz.br

Table of Contents

1.Jupyter Notebook with the codes for training machine learning models…….....……………………. S3

2.Ranking of hyperparameters of SVM model……………………………………..……………………. S19

1. **Jupyter Notebook with the codes for training machine learning models**

```
!python --version

Python 3.10.12
```

Code Explanation

Library Imports

Libraries such as Pandas. NumPy. and scikit-learn are imported for data manipulation. numerical operations. and machine learning functionalities. Seaborn and Matplotlib are imported for visualization purposes.

Data Preprocessing

Assuming there's some data preprocessing steps done before this point such as loading data into a DataFrame and handling missing values.

Feature Scaling

MinMaxScaler from scikit-learn is used to scale the features to a range between 0 and 1.

Handling Class Imbalance

RandomUnderSampler from imbalanced-learn is used to handle class imbalance by undersampling the majority class.

Splitting Data into Train and Test Sets

train_test_split from scikit-learn is used to split the resampled data into training and testing sets.

Model Training

A Support Vector Machine (SVM) model is trained using the SVC class from scikit-learn.

Model Evaluation Metrics

Precision. recall. F1-score. accuracy. and mean squared error are calculated using appropriate functions from scikit-learn.

Hyperparameter Tuning

GridSearchCV from scikit-learn is used for hyperparameter tuning of the SVM model.

Visualization

A confusion matrix heatmap is plotted using Seaborn and Matplotlib to visualize the performance of the trained model.

```
import pandas as pd
import numpy as np
```

```
from collections import Counter
from sklearn.metrics import precision_score
from sklearn.metrics import recall_score
from sklearn.metrics import f1_score
from sklearn.metrics import accuracy_score
import seaborn as sns
import matplotlib.pyplot as plt
from sklearn.svm import SVC
from sklearn.linear_model import LogisticRegression
from sklearn.preprocessing import MinMaxScaler
from sklearn.model_selection import train_test_split
from imblearn.under_sampling import RandomUnderSampler
from sklearn.model_selection import GridSearchCV, StratifiedKFold
from sklearn.metrics import mean_squared_error
```

Reading Data

Two Excel files. "CompiledRuns.xlsx" and "MeltingPoints.xlsx". are read into Pandas DataFrames (`dfResults` and `dfMelting` respectively) using the `pd.read_excel()` function.

Filtering Data

1. `dfResults` is filtered to include only rows where the value in the "Run" column is greater than or equal to 6.
2. From the filtered `dfResults`. a new DataFrame `dfResultsFiltered` is created containing only the "ConcatName" and "Result" columns.

Data Exploration

1. `dfResultsFiltered.shape`: Returns the shape (number of rows and columns) of the DataFrame `dfResultsFiltered`.
2. `dfResultsFiltered.isnull().sum()`: Returns the sum of missing values in each column of the DataFrame `dfResultsFiltered`.
3. `dfResultsFiltered.value_counts("Result")`: Returns the count of unique values in the "Result" column of the DataFrame `dfResultsFiltered`.

```
dfResults = pd.read_excel("CompiledRuns.xlsx",sheet_name="Results")
dfMelting = pd.read_excel("MeltingPoints.xlsx",sheet_name="Points")

dfResults = dfResults[dfResults['Run'] >=6]

dfResultsFiltered = dfResults[["ConcatName", "Result"]]

dfResultsFiltered.shape

(1724, 2)

dfResultsFiltered.isnull().sum()

ConcatName    0
Result        0
dtype: int64
```

```
dfResultsFiltered.value_counts("Result")
```

```
Result
1.0     634
2.0     560
0.0     530
dtype: int64
```

Code Explanation

Creating Sequential Observations

A new column named "Observation" is added to the DataFrame `dfMelting`. This column represents the sequential count of observations within each group defined by the "ConcatName" column.

Pivot Table

A pivot table is created from the DataFrame `dfMelting`. The index of the pivot table is set to "ConcatName". the columns are set to "Observation". and the values are extracted from the "Derivative" column.

Renaming Columns

The columns of the pivot table (`df_pivot`) are renamed using list comprehension. where each column is given a formatted name.

```
dfMelting['Observation'] = dfMelting.groupby('ConcatName').cumcount()
+ 1


df_pivot = dfMelting.pivot(index='ConcatName', columns='Observation',
values='Derivative')


df_pivot.columns = [f'{col}' for col in df_pivot.columns];df_pivot
```

{"type":"dataframe","variable_name":"df_pivot"}

Merging DataFrames

Two DataFrames. `dfResultsFiltered` and `df_pivot`. are merged using the `pd.merge()` function. The merge is performed on the "ConcatName" column. with a left join specified using the `how='left'` parameter.

```
df_merged = pd.merge(dfResultsFiltered, df_pivot, on='ConcatName',
how='left')

df_merged.head()
```

{"type":"dataframe","variable_name":"df_merged"}

```
df_merged.shape
```

```
(1724, 194)
```

Checking for Missing Values

The `isnull().sum()` function is used to calculate the sum of missing values in each column of the DataFrame `df_merged`.

Filtering Rows with Missing Values

The DataFrame `df_merged` is filtered to include only rows where the value in the column '192' is missing. using the `pd.isna()` function.

```
df_merged.isnull().sum()
```

```
ConcatName      0
Result          0
1               0
2               0
3               0
             ...
188             0
189             0
190             0
191             0
192           475
Length: 194, dtype: int64
```

```
df_merged[pd.isna(df_merged['192'])]
```

{"type":"dataframe"}

Dropping Columns

The column labeled '192' is dropped from the DataFrame `df_merged` using the `drop()` function.

```
df_merged = df_merged.drop(columns=['192'])
```

Splitting Target and Features

- `y = df_merged['Result']`: Creates a Series `y` containing the target variable 'Result' from the DataFrame `df_merged`.
- `X = df_merged.drop(['ConcatName'. 'Result']. axis=1)`: Creates a DataFrame `X` containing the features by dropping the columns 'ConcatName' and 'Result' from `df_merged`.

Splitting Data into Train and Test Sets

- `X_train. X_test. y_train. y_test = train_test_split(X. y. random_state=0)`: Splits the features and target variables into training and testing sets. `X_train` and `X_test` contain the feature values for training and testing respectively. while `y_train` and `y_test` contain the corresponding target values. The `random_state=0` parameter ensures reproducibility by fixing the random seed.

Feature Scaling

- `scaler = MinMaxScaler()`: Initializes a MinMaxScaler object. which is used to scale features to a specific range (by default. between 0 and 1).
- `X_train_normalized = scaler.fit_transform(X_train)`: Fits the scaler to the training data (`X_train`) and then transforms it to normalized values.
- `X_test_normalized = scaler.transform(X_test)`: Uses the scaler fitted on the training data to transform the test data (`X_test`) to normalized values. It's important to note that the same scaler instance is used for both training and testing sets to ensure consistency in scaling.

```
y = df_merged['Result']

X = df_merged.drop(['ConcatName', 'Result'], axis=1)


# BE.9: 0
# Non-BE.9: 1
# Inconclusive: 2

X_train_eval, X_test, y_train_eval, y_test = train_test_split(
    X, y, random_state=0, test_size=0.20)

X_train, X_eval, y_train, y_eval = train_test_split(
    X_train_eval, y_train_eval, random_state=0, test_size=0.25)

scaler = MinMaxScaler()

X_train_normalized = scaler.fit_transform(X_train)
X_eval_normalized = scaler.transform(X_eval)
X_test_normalized = scaler.transform(X_test)
```

Resampling with Random Under-sampling

- `under_sampler = RandomUnderSampler(random_state=42)`: Initializes a RandomUnderSampler object with a fixed random state of 42. Random under-sampling is a technique used to balance imbalanced datasets by randomly removing samples from the majority class.
- `X_res_normalized. y_res_normalized = under_sampler.fit_resample(X_train_normalized. y_train)`: Applies random under-sampling to the normalized training data (`X_train_normalized`) and corresponding target variable (`y_train`). The `fit_resample()` method fits the under-sampler to the training data and then applies the resampling technique to balance the

classes. The resulting `X_res_normalized` and `y_res_normalized` contain the resampled feature and target data respectively.

```
under_sampler = RandomUnderSampler(random_state=42)

X_res_normalized, y_res_normalized =
under_sampler.fit_resample(X_train_normalized, y_train)

print(X_res_normalized.shape)
print(X_eval_normalized.shape)
print(X_test_normalized.shape)

(948, 191)
(345, 191)
(345, 191)
```

Support Vector Machine (SVM)

- `svm_model = SVC(kernel='linear')`: Initializes a Support Vector Machine (SVM) model with a linear kernel. SVM is a supervised learning algorithm used for classification and regression tasks.
- `svm_model.fit(X_res_normalized. y_res_normalized)`: Trains the SVM model on the resampled and normalized training data (`X_res_normalized`) and corresponding target variable (`y_res_normalized`).
- `y_pred_svm = svm_model.predict(X_test_normalized)`: Predicts the target variable for the normalized test data (`X_test_normalized`) using the trained SVM model.

Logistic Regression

- `logistic_model = LogisticRegression()`: Initializes a logistic regression model. Logistic regression is a classification algorithm used to model the probability of a outcome.
- `logistic_model.fit(X_res_normalized. y_res_normalized)`: Trains the logistic regression model on the resampled and normalized training data (`X_res_normalized`) and corresponding target variable (`y_res_normalized`).
- `y_pred_reg = logistic_model.predict(X_test_normalized)`: Predicts the target variable for the normalized test data (`X_test_normalized`) using the trained logistic regression model.

Gradient Boosting

- `from xgboost import XGBClassifier`: Imports the XGBoost classifier from the XGBoost library. XGBoost is an implementation of gradient boosting algorithms.
- `model = XGBClassifier(n_jobs=-1)`: Initializes an XGBoost classifier with all available CPU cores (`n_jobs=-1`).
- `model.fit(X_res_normalized. y_res_normalized)`: Trains the XGBoost classifier on the resampled and normalized training data (`X_res_normalized`) and corresponding target variable (`y_res_normalized`).

- `y_pred_gb = model.predict(X_test_normalized)`: Predicts the target variable for the normalized test data (`X_test_normalized`) using the trained XGBoost classifier.

```
# SVM

svm_model = SVC(kernel='linear')

svm_model.fit(X_res_normalized, y_res_normalized)

y_pred_svm = svm_model.predict(X_eval_normalized)

# Logistic regression

logistic_model = LogisticRegression()

logistic_model.fit(X_res_normalized, y_res_normalized)

y_pred_reg = logistic_model.predict(X_eval_normalized)

# GradientBoosting

from xgboost import XGBClassifier

model = XGBClassifier(n_jobs = -1)

model.fit(X_res_normalized, y_res_normalized)

y_pred_gb = model.predict(X_eval_normalized)
```

Support Vector Machine (SVM) Metrics

- `print('Precision score %s' % precision_score(y_test. y_pred_svm. average=None))`: Computes and prints the precision score for each class separately using the `precision_score` function.
- `print('Recall score %s' % recall_score(y_test. y_pred_svm. average=None))`: Computes and prints the recall score for each class separately using the `recall_score` function.
- `print('F1-score score %s' % f1_score(y_test. y_pred_svm. average=None))`: Computes and prints the F1-score for each class separately using the `f1_score` function.
- `print('Accuracy score %s' % accuracy_score(y_test. y_pred_svm))`: Computes and prints the accuracy score using the `accuracy_score` function.

Logistic Regression Metrics

- Similar to SVM. these lines of code compute and print precision. recall. F1-score. and accuracy metrics for the logistic regression model (`y_pred_reg`).

Gradient Boosting Metrics

- Similar to SVM and logistic regression. these lines of code compute and print precision. recall. F1-score. and accuracy metrics for the gradient boosting model (`y_pred_gb`).

```
# SVM

print('Precision score %s' % precision_score(y_eval. y_pred_svm.
average=None))
print('Recall score %s' % recall_score(y_eval. y_pred_svm.
average=None))
print('F1-score score %s' % f1_score(y_eval. y_pred_svm.
average=None))
print('Accuracy score %s' % accuracy_score(y_eval. y_pred_svm))
```

```
Precision score [0.99130435 0.99173554 0.93577982]
Recall score [0.97435897 0.96774194 0.98076923]
F1-score score [0.98275862 0.97959184 0.95774648]
Accuracy score 0.9739130434782609
```

```
# Logistic Regression

print('Precision score %s' % precision_score(y_eval. y_pred_reg.
average=None))
print('Recall score %s' % recall_score(y_eval. y_pred_reg.
average=None))
print('F1-score score %s' % f1_score(y_eval. y_pred_reg.
average=None))
print('Accuracy score %s' % accuracy_score(y_eval. y_pred_reg))
```

```
Precision score [1.         0.976      0.90990991]
Recall score [0.93162393 0.98387097 0.97115385]
F1-score score [0.96460177 0.97991968 0.93953488]
Accuracy score 0.9623188405797102
```

```
# GradientBoosting

print('Precision score %s' % precision_score(y_eval. y_pred_gb.
average=None))
print('Recall score %s' % recall_score(y_eval. y_pred_gb.
average=None))
print('F1-score score %s' % f1_score(y_eval. y_pred_gb. average=None))
print('Accuracy score %s' % accuracy_score(y_eval. y_pred_gb))
```

```
Precision score [0.98290598 0.976      0.96116505]
Recall score [0.98290598 0.98387097 0.95192308]
F1-score score [0.98290598 0.97991968 0.95652174]
Accuracy score 0.9739130434782609
```

Results Dictionary

A dictionary named `results` is created to store the evaluation metrics for different algorithms. Each key in the dictionary corresponds to a different metric. and the values are lists containing the metric values for each algorithm.

Creating DataFrame

The dictionary `results` is used to create a Pandas DataFrame named `df_results`. Each key in the dictionary becomes a column in the DataFrame.

Saving to File

The DataFrame `df_results` is saved to a tab-separated values (TSV) file named "AlgorithmComparison.tsv" using the `to_csv()` function. The parameter `sep='\t'` specifies that tabs should be used as separators between values. and `index=False` ensures that the DataFrame index is not included in the output file.

```
results = {
    'Algorithm': ['SVM'. 'Logistic Regression'. 'GradientBoosting'].
    'Precision': [
        precision_score(y_eval. y_pred_svm. average='micro').
        precision_score(y_eval. y_pred_reg. average='micro').
        precision_score(y_eval. y_pred_gb. average='micro')
    ].
    'Recall': [
        recall_score(y_eval. y_pred_svm. average='micro').
        recall_score(y_eval. y_pred_reg. average='micro').
        recall_score(y_eval. y_pred_gb. average='micro')
    ].
    'F1-score': [
        f1_score(y_eval. y_pred_svm. average='micro').
        f1_score(y_eval. y_pred_reg. average='micro').
        f1_score(y_eval. y_pred_gb. average='micro')
    ].
    'Accuracy': [
        accuracy_score(y_eval. y_pred_svm).
        accuracy_score(y_eval. y_pred_reg).
        accuracy_score(y_eval. y_pred_gb)
    ]
}

df_results = pd.DataFrame(results)


df_results.to_csv('AlgorithmComparison.tsv'.sep='\t'.index=False)
```

Function Definition

- The function `plotMatrix` is defined to visualize the confusion matrix for classification models.
- It takes three arguments: `y_pred` (predicted labels). `labels` (list of class labels. defaulting to ["BE.9". "Non-BE.9". "Inconclusive"]). and `figName` (name of the figure file to be saved. defaulting to "ConfusionMatrix").

Confusion Matrix Computation

- Inside the function. the confusion matrix is computed using the predicted labels (`y_pred`) and the actual labels (`y_test`).
- The confusion matrix is then converted to percentages by dividing each element by the sum of its corresponding row.

Plot Configuration

- The function configures the heatmap plot using Seaborn and Matplotlib libraries.
- For each cell in the heatmap. text is added to display the percentage and count of samples. with text color set based on the percentage value.

Saving and Displaying Plot

- The plot is saved as a PNG image with the filename specified by `figName`.
- The plot is displayed using Matplotlib's `plt.show()` function.

```
import numpy as np
import seaborn as sns
import matplotlib.pyplot as plt
from sklearn.metrics import confusion_matrix

def plotMatrix(y_test. y_pred. labels = ["BE.9". "Non-BE.9".
"Inconclusive"]. figName = "ConfusionMatrix"):

  conf_matrix = confusion_matrix(y_test. y_pred)
  conf_matrix_percent = conf_matrix / np.sum(conf_matrix. axis=1.
keepdims=True)

  # Configuração do gráfico
  class_names = labels
  sns.set(font_scale=1.2)
  plt.figure(figsize=(8. 6))
  sns.heatmap(conf_matrix_percent. annot=False. fmt="d". cmap="Blues".
xticklabels=class_names. yticklabels=class_names. cbar=False)
  for i in range(len(class_names)):
      for j in range(len(class_names)):
          if conf_matrix_percent[i. j] > 0.7:
            color = "white"
          else:
            color="black"
          plt.text(j + 0.5. i + 0.5. f"{conf_matrix_percent[i.
j]:.2%}\n({conf_matrix[i. j]})". ha="center". va="center".
color=color)

  plt.xlabel('Predicted Values')
  plt.ylabel('Real Values')
  plt.savefig(f'{figName}.png'. dpi=300)
  plt.show()

plotMatrix(y_eval. y_pred_svm. figName = "svmMatrix")
```

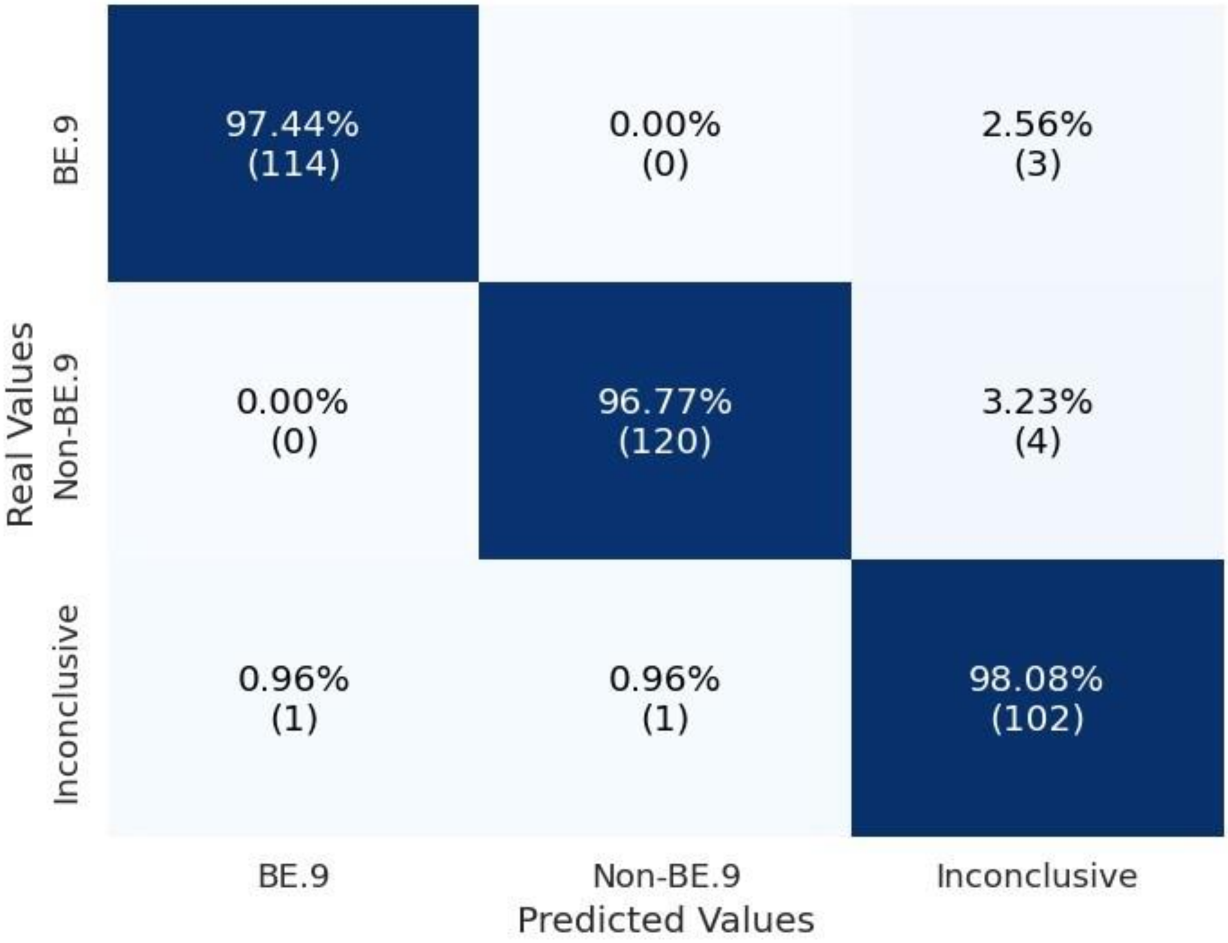

Imports

- The necessary libraries such as Pandas. SVC (Support Vector Classifier) from scikit-learn. GridSearchCV for hyperparameter tuning. and various metrics from scikit-learn are imported.

Creating Results DataFrame

- A DataFrame named `results_df` is created to store the results of different SVM models with various hyperparameters. It includes columns for parameters. mean squared error (MSE). precision. recall. F1-score. and accuracy.

Default SVM Model

- A default SVM model (`svm_model_standard`) is trained using the default parameters and evaluated on the test set to obtain baseline performance metrics such as MSE. precision. recall. F1-score. and accuracy.

Parameter Grid

- A parameter grid (`param_grid`) is defined. specifying various values for the hyperparameters 'C' (regularization parameter). 'kernel' (type of kernel function). 'gamma' (kernel coefficient). and 'degree' (degree of the polynomial kernel).

Grid Search

- GridSearchCV is used to perform hyperparameter tuning for the SVM model. It searches through different combinations of hyperparameters specified in `param_grid` to find the combination that optimizes the accuracy metric.

Model Evaluation

- For each combination of hyperparameters tested during the grid search. a new SVM model is trained using those parameters. and its performance is evaluated on the test set. Metrics such as MSE. precision. recall. F1-score. and accuracy are computed for each model.

Saving Results

- The results. including hyperparameters and evaluation metrics. are stored in the DataFrame `results_df`.
- The DataFrame is saved to an Excel file named "results_svm.xlsx" using the `to_excel()` function for further analysis.

```
import pandas as pd
from sklearn.svm import SVC
from sklearn.model_selection import GridSearchCV
from sklearn.metrics import mean_squared_error. precision_score.
recall_score. f1_score. accuracy_score
from sklearn.model_selection import StratifiedKFold

# Creating a DataFrame to store the results
results_df = pd.DataFrame(columns=['Parameters'. 'Precision'.
'Recall'. 'F1'. 'Accuracy'])

# Default SVM Configuration
svm_model_standard = SVC()
svm_model_standard.fit(X_res_normalized. y_res_normalized)
y_pred_standard = svm_model_standard.predict(X_eval_normalized)

# Evaluating the default model on the test set
precision_standard = precision_score(y_eval. y_pred_standard.
average='micro')
recall_standard = recall_score(y_eval. y_pred_standard.
average='micro')
f1_standard = f1_score(y_eval. y_pred_standard. average='micro')
accuracy_standard = accuracy_score(y_eval. y_pred_standard)

# Adding default results to the DataFrame
results_df = results_df.append({
    'Parameters': 'Default'.
    'Precision': precision_standard.
    'Recall': recall_standard.
    'F1': f1_standard.
    'Accuracy': accuracy_standard
}. ignore_index=True)
```

```
# Defining the parameters you want to test
param_grid = {
    'C': [0.001. 0.01. 0.1. 1. 10. 100. 1000].
    'kernel': ['linear'. 'poly'. 'rbf'].
    'gamma': [0.01. 0.1. 1. 'auto'].
    'degree': [2. 3. 4].
}

# Creating a GridSearchCV object
grid_search = GridSearchCV(SVC(). param_grid. scoring='accuracy'.
cv=StratifiedKFold(n_splits=5). verbose=1. n_jobs=-1)

# Running the grid search
grid_search.fit(X_res_normalized. y_res_normalized)

# Iterating over all tested parameter combinations
for i. params in enumerate(grid_search.cv_results_['params']):
    # Creating an instance of the SVC model with the best parameters
    best_model = SVC(**params)

    # Training the model with the best parameters
    best_model.fit(X_res_normalized. y_res_normalized)

    # Evaluating the best model on the test set
    y_pred = best_model.predict(X_eval_normalized)
    precision = precision_score(y_eval. y_pred. average='micro')
    recall = recall_score(y_eval. y_pred. average='micro')
    f1 = f1_score(y_eval. y_pred. average='micro')
    accuracy = accuracy_score(y_eval. y_pred)

    # Adding grid search results to the DataFrame
    results_df = results_df.append({
        'Parameters': params.
        'Precision': precision.
        'Recall': recall.
        'F1': f1.
        'Accuracy': accuracy
    }. ignore_index=True)

# Saving the results to an Excel file
results_df.to_excel('results_svm.xlsx'. index=False)
```

Training the Model

- The code begins by training the SVM model with the best hyperparameters.
- An SVM model for classification is created with the following hyperparameters:
  - Kernel: Radial Basis Function (RBF). specified by `kernel='rbf'`.

- Gamma: Auto. specified by `gamma="auto"`. Gamma defines how far the influence of a single training example reaches. with a higher value indicating a tighter fit.
- Degree: 2. specified by `degree=2`. Degree is the degree of the polynomial kernel function (only relevant if `kernel='poly'`).
- Regularization Parameter (C): 100. specified by `C=100`. C controls the trade-off between a smooth decision boundary and classifying the training points correctly.

- The created SVM model is stored in the variable `svm_modelTunned`.

Model Training

- The created SVM model (`svm_modelTunned`) is trained on the normalized and resampled training data (`X_res_normalized`. `y_res_normalized`) using the `fit()` method.

Making Predictions

- Once the model is trained. predictions are made on the normalized test set (`X_test_normalized`) using the `predict()` method.
- The predicted labels are stored in the variable `y_pred_svmTunned`.

```
# SVM

# Training the model with the best hyperparameters
# Creating an SVM model for classification
svm_modelTunned = SVC(kernel='rbf'. gamma="auto". degree=2. C=100)
# Training the model
svm_modelTunned.fit(X_res_normalized. y_res_normalized)

# Making predictions on the eval set
y_pred_eval_svmTunned = svm_modelTunned.predict(X_eval_normalized)

# Making predictions on the test set
y_pred_svmTunned = svm_modelTunned.predict(X_test_normalized)

# SVM

print('Precision score %s' % precision_score(y_eval.
y_pred_eval_svmTunned. average=None))
print('Recall score %s' % recall_score(y_eval. y_pred_eval_svmTunned.
average=None))
print('F1-score score %s' % f1_score(y_eval. y_pred_eval_svmTunned.
average=None))
print('Accuracy score %s' % accuracy_score(y_eval.
y_pred_eval_svmTunned))

Precision score [1.         0.96850394 0.98039216]
Recall score [0.99145299 0.99193548 0.96153846]
F1-score score [0.99570815 0.98007968 0.97087379]
Accuracy score 0.9826086956521739
```

```
# SVM

print('Precision score %s' % precision_score(y_test, y_pred_svmTunned, average=None))
print('Recall score %s' % recall_score(y_test, y_pred_svmTunned, average=None))
print('F1-score score %s' % f1_score(y_test, y_pred_svmTunned, average=None))
print('Accuracy score %s' % accuracy_score(y_test, y_pred_svmTunned))
```

```
Precision score [0.98924731 0.95714286 0.95535714]
Recall score [0.94845361 1.         0.93859649]
F1-score score [0.96842105 0.97810219 0.94690265]
Accuracy score 0.9652173913043478
```

```
plotMatrix(y_test, y_pred_svmTunned, figName="fineTunnedSVM")
```

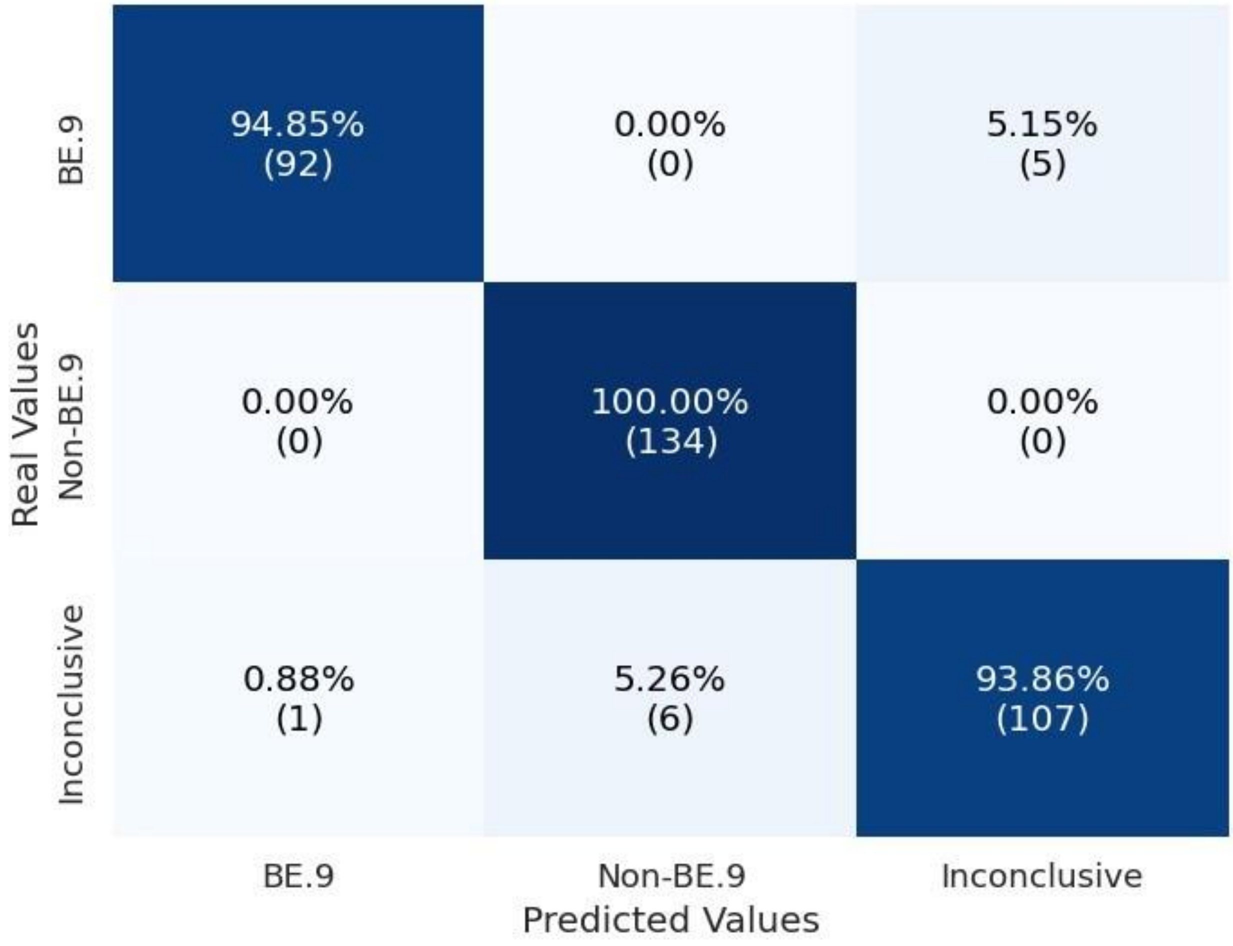

Saving the Model

- The `joblib.dump()` function is used to save the trained model.
- The model to be saved is specified as the first argument (`model`).

- The second argument is the file path where the model will be saved (`'/content/drive/MyDrive/Machine Learning/TR/model.joblib'`).
- This file path should include the desired location and filename for the saved model.

Note

- It's essential to ensure that the directory specified for saving the model exists and that the file path is accessible.

```
import joblib

# Saving the model
joblib.dump(model, '/content/model.joblib')
```

```
['/content/model.joblib']
```

2. Ranking of hyperparameters of SVM model

Parameters	Precision	Recall	F1	Accuracy
Default	0.976811594	0.976811594	0.976811594	0.976811594
{'C': 0.001. 'degree': 2. 'gamma': 0.01. 'kernel': 'linear'}	0.811594203	0.811594203	0.811594203	0.811594203
{'C': 0.001. 'degree': 2. 'gamma': 0.01. 'kernel': 'poly'}	0.4	0.4	0.4	0.4
{'C': 0.001. 'degree': 2. 'gamma': 0.01. 'kernel': 'rbf'}	0.75942029	0.75942029	0.75942029	0.75942029
{'C': 0.001. 'degree': 2. 'gamma': 0.1. 'kernel': 'linear'}	0.811594203	0.811594203	0.811594203	0.811594203
{'C': 0.001. 'degree': 2. 'gamma': 0.1. 'kernel': 'poly'}	0.423188406	0.423188406	0.423188406	0.423188406
{'C': 0.001. 'degree': 2. 'gamma': 0.1. 'kernel': 'rbf'}	0.855072464	0.855072464	0.855072464	0.855072464
{'C': 0.001. 'degree': 2. 'gamma': 1. 'kernel': 'linear'}	0.811594203	0.811594203	0.811594203	0.811594203
{'C': 0.001. 'degree': 2. 'gamma': 1. 'kernel': 'poly'}	0.889855072	0.889855072	0.889855072	0.889855072
{'C': 0.001. 'degree': 2. 'gamma': 1. 'kernel': 'rbf'}	0.866666667	0.866666667	0.866666667	0.866666667
{'C': 0.001. 'degree': 2. 'gamma': 'auto'. 'kernel': 'linear'}	0.811594203	0.811594203	0.811594203	0.811594203
{'C': 0.001. 'degree': 2. 'gamma': 'auto'. 'kernel': 'poly'}	0.4	0.4	0.4	0.4
{'C': 0.001. 'degree': 2. 'gamma': 'auto'. 'kernel': 'rbf'}	0.733333333	0.733333333	0.733333333	0.733333333
{'C': 0.001. 'degree': 3. 'gamma': 0.01. 'kernel': 'linear'}	0.811594203	0.811594203	0.811594203	0.811594203
{'C': 0.001. 'degree': 3. 'gamma': 0.01. 'kernel': 'poly'}	0.336231884	0.336231884	0.336231884	0.336231884
{'C': 0.001. 'degree': 3. 'gamma': 0.01. 'kernel': 'rbf'}	0.75942029	0.75942029	0.75942029	0.75942029
{'C': 0.001. 'degree': 3. 'gamma': 0.1. 'kernel': 'linear'}	0.811594203	0.811594203	0.811594203	0.811594203
{'C': 0.001. 'degree': 3. 'gamma': 0.1. 'kernel': 'poly'}	0.605797101	0.605797101	0.605797101	0.605797101
{'C': 0.001. 'degree': 3. 'gamma': 0.1. 'kernel': 'rbf'}	0.855072464	0.855072464	0.855072464	0.855072464
{'C': 0.001. 'degree': 3. 'gamma': 1. 'kernel': 'linear'}	0.811594203	0.811594203	0.811594203	0.811594203
{'C': 0.001. 'degree': 3. 'gamma': 1. 'kernel': 'poly'}	0.930434783	0.930434783	0.930434783	0.930434783
{'C': 0.001. 'degree': 3. 'gamma': 1. 'kernel': 'rbf'}	0.866666667	0.866666667	0.866666667	0.866666667
{'C': 0.001. 'degree': 3. 'gamma': 'auto'. 'kernel': 'linear'}	0.811594203	0.811594203	0.811594203	0.811594203
{'C': 0.001. 'degree': 3. 'gamma': 'auto'. 'kernel': 'poly'}	0.336231884	0.336231884	0.336231884	0.336231884
{'C': 0.001. 'degree': 3. 'gamma': 'auto'. 'kernel': 'rbf'}	0.733333333	0.733333333	0.733333333	0.733333333
{'C': 0.001. 'degree': 4. 'gamma': 0.01. 'kernel': 'linear'}	0.811594203	0.811594203	0.811594203	0.811594203
{'C': 0.001. 'degree': 4. 'gamma': 0.01. 'kernel': 'poly'}	0.315942029	0.315942029	0.315942029	0.315942029
{'C': 0.001. 'degree': 4. 'gamma': 0.01. 'kernel': 'rbf'}	0.75942029	0.75942029	0.75942029	0.75942029
{'C': 0.001. 'degree': 4. 'gamma': 0.1. 'kernel': 'linear'}	0.811594203	0.811594203	0.811594203	0.811594203
{'C': 0.001. 'degree': 4. 'gamma': 0.1. 'kernel': 'poly'}	0.684057971	0.684057971	0.684057971	0.684057971
{'C': 0.001. 'degree': 4. 'gamma': 0.1. 'kernel': 'rbf'}	0.855072464	0.855072464	0.855072464	0.855072464
{'C': 0.001. 'degree': 4. 'gamma': 1. 'kernel': 'linear'}	0.811594203	0.811594203	0.811594203	0.811594203
{'C': 0.001. 'degree': 4. 'gamma': 1. 'kernel': 'poly'}	0.950724638	0.950724638	0.950724638	0.950724638
{'C': 0.001. 'degree': 4. 'gamma': 1. 'kernel': 'rbf'}	0.866666667	0.866666667	0.866666667	0.866666667
{'C': 0.001. 'degree': 4. 'gamma': 'auto'. 'kernel': 'linear'}	0.811594203	0.811594203	0.811594203	0.811594203
{'C': 0.001. 'degree': 4. 'gamma': 'auto'. 'kernel': 'poly'}	0.315942029	0.315942029	0.315942029	0.315942029
{'C': 0.001. 'degree': 4. 'gamma': 'auto'. 'kernel': 'rbf'}	0.733333333	0.733333333	0.733333333	0.733333333
{'C': 0.01. 'degree': 2. 'gamma': 0.01. 'kernel': 'linear'}	0.915942029	0.915942029	0.915942029	0.915942029
{'C': 0.01. 'degree': 2. 'gamma': 0.01. 'kernel': 'poly'}	0.4	0.4	0.4	0.4
{'C': 0.01. 'degree': 2. 'gamma': 0.01. 'kernel': 'rbf'}	0.75942029	0.75942029	0.75942029	0.75942029
{'C': 0.01. 'degree': 2. 'gamma': 0.1. 'kernel': 'linear'}	0.915942029	0.915942029	0.915942029	0.915942029

{'C': 0.01. 'degree': 2. 'gamma': 0.1. 'kernel': 'poly'}	0.805797101	0.805797101	0.805797101	0.805797101
{'C': 0.01. 'degree': 2. 'gamma': 0.1. 'kernel': 'rbf'}	0.855072464	0.855072464	0.855072464	0.855072464
{'C': 0.01. 'degree': 2. 'gamma': 1. 'kernel': 'linear'}	0.915942029	0.915942029	0.915942029	0.915942029
{'C': 0.01. 'degree': 2. 'gamma': 1. 'kernel': 'poly'}	0.953623188	0.953623188	0.953623188	0.953623188
{'C': 0.01. 'degree': 2. 'gamma': 1. 'kernel': 'rbf'}	0.866666667	0.866666667	0.866666667	0.866666667
{'C': 0.01. 'degree': 2. 'gamma': 'auto'. 'kernel': 'linear'}	0.915942029	0.915942029	0.915942029	0.915942029
{'C': 0.01. 'degree': 2. 'gamma': 'auto'. 'kernel': 'poly'}	0.4	0.4	0.4	0.4
{'C': 0.01. 'degree': 2. 'gamma': 'auto'. 'kernel': 'rbf'}	0.733333333	0.733333333	0.733333333	0.733333333
{'C': 0.01. 'degree': 3. 'gamma': 0.01. 'kernel': 'linear'}	0.915942029	0.915942029	0.915942029	0.915942029
{'C': 0.01. 'degree': 3. 'gamma': 0.01. 'kernel': 'poly'}	0.336231884	0.336231884	0.336231884	0.336231884
{'C': 0.01. 'degree': 3. 'gamma': 0.01. 'kernel': 'rbf'}	0.75942029	0.75942029	0.75942029	0.75942029
{'C': 0.01. 'degree': 3. 'gamma': 0.1. 'kernel': 'linear'}	0.915942029	0.915942029	0.915942029	0.915942029
{'C': 0.01. 'degree': 3. 'gamma': 0.1. 'kernel': 'poly'}	0.814492754	0.814492754	0.814492754	0.814492754
{'C': 0.01. 'degree': 3. 'gamma': 0.1. 'kernel': 'rbf'}	0.855072464	0.855072464	0.855072464	0.855072464
{'C': 0.01. 'degree': 3. 'gamma': 1. 'kernel': 'linear'}	0.915942029	0.915942029	0.915942029	0.915942029
{'C': 0.01. 'degree': 3. 'gamma': 1. 'kernel': 'poly'}	0.962318841	0.962318841	0.962318841	0.962318841
{'C': 0.01. 'degree': 3. 'gamma': 1. 'kernel': 'rbf'}	0.866666667	0.866666667	0.866666667	0.866666667
{'C': 0.01. 'degree': 3. 'gamma': 'auto'. 'kernel': 'linear'}	0.915942029	0.915942029	0.915942029	0.915942029
{'C': 0.01. 'degree': 3. 'gamma': 'auto'. 'kernel': 'poly'}	0.336231884	0.336231884	0.336231884	0.336231884
{'C': 0.01. 'degree': 3. 'gamma': 'auto'. 'kernel': 'rbf'}	0.733333333	0.733333333	0.733333333	0.733333333
{'C': 0.01. 'degree': 4. 'gamma': 0.01. 'kernel': 'linear'}	0.915942029	0.915942029	0.915942029	0.915942029
{'C': 0.01. 'degree': 4. 'gamma': 0.01. 'kernel': 'poly'}	0.315942029	0.315942029	0.315942029	0.315942029
{'C': 0.01. 'degree': 4. 'gamma': 0.01. 'kernel': 'rbf'}	0.75942029	0.75942029	0.75942029	0.75942029
{'C': 0.01. 'degree': 4. 'gamma': 0.1. 'kernel': 'linear'}	0.915942029	0.915942029	0.915942029	0.915942029
{'C': 0.01. 'degree': 4. 'gamma': 0.1. 'kernel': 'poly'}	0.814492754	0.814492754	0.814492754	0.814492754
{'C': 0.01. 'degree': 4. 'gamma': 0.1. 'kernel': 'rbf'}	0.855072464	0.855072464	0.855072464	0.855072464
{'C': 0.01. 'degree': 4. 'gamma': 1. 'kernel': 'linear'}	0.915942029	0.915942029	0.915942029	0.915942029
{'C': 0.01. 'degree': 4. 'gamma': 1. 'kernel': 'poly'}	0.962318841	0.962318841	0.962318841	0.962318841
{'C': 0.01. 'degree': 4. 'gamma': 1. 'kernel': 'rbf'}	0.866666667	0.866666667	0.866666667	0.866666667
{'C': 0.01. 'degree': 4. 'gamma': 'auto'. 'kernel': 'linear'}	0.915942029	0.915942029	0.915942029	0.915942029
{'C': 0.01. 'degree': 4. 'gamma': 'auto'. 'kernel': 'poly'}	0.315942029	0.315942029	0.315942029	0.315942029
{'C': 0.01. 'degree': 4. 'gamma': 'auto'. 'kernel': 'rbf'}	0.733333333	0.733333333	0.733333333	0.733333333
{'C': 0.1. 'degree': 2. 'gamma': 0.01. 'kernel': 'linear'}	0.95942029	0.95942029	0.95942029	0.95942029
{'C': 0.1. 'degree': 2. 'gamma': 0.01. 'kernel': 'poly'}	0.423188406	0.423188406	0.423188406	0.423188406
{'C': 0.1. 'degree': 2. 'gamma': 0.01. 'kernel': 'rbf'}	0.857971014	0.857971014	0.857971014	0.857971014
{'C': 0.1. 'degree': 2. 'gamma': 0.1. 'kernel': 'linear'}	0.95942029	0.95942029	0.95942029	0.95942029
{'C': 0.1. 'degree': 2. 'gamma': 0.1. 'kernel': 'poly'}	0.889855072	0.889855072	0.889855072	0.889855072
{'C': 0.1. 'degree': 2. 'gamma': 0.1. 'kernel': 'rbf'}	0.91884058	0.91884058	0.91884058	0.91884058
{'C': 0.1. 'degree': 2. 'gamma': 1. 'kernel': 'linear'}	0.95942029	0.95942029	0.95942029	0.95942029
{'C': 0.1. 'degree': 2. 'gamma': 1. 'kernel': 'poly'}	0.971014493	0.971014493	0.971014493	0.971014493
{'C': 0.1. 'degree': 2. 'gamma': 1. 'kernel': 'rbf'}	0.939130435	0.939130435	0.939130435	0.939130435
{'C': 0.1. 'degree': 2. 'gamma': 'auto'. 'kernel': 'linear'}	0.95942029	0.95942029	0.95942029	0.95942029
{'C': 0.1. 'degree': 2. 'gamma': 'auto'. 'kernel': 'poly'}	0.4	0.4	0.4	0.4
{'C': 0.1. 'degree': 2. 'gamma': 'auto'. 'kernel': 'rbf'}	0.811594203	0.811594203	0.811594203	0.811594203

{'C': 0.1. 'degree': 3. 'gamma': 0.01. 'kernel': 'linear'}	0.95942029	0.95942029	0.95942029	0.95942029
{'C': 0.1. 'degree': 3. 'gamma': 0.01. 'kernel': 'poly'}	0.336231884	0.336231884	0.336231884	0.336231884
{'C': 0.1. 'degree': 3. 'gamma': 0.01. 'kernel': 'rbf'}	0.857971014	0.857971014	0.857971014	0.857971014
{'C': 0.1. 'degree': 3. 'gamma': 0.1. 'kernel': 'linear'}	0.95942029	0.95942029	0.95942029	0.95942029
{'C': 0.1. 'degree': 3. 'gamma': 0.1. 'kernel': 'poly'}	0.875362319	0.875362319	0.875362319	0.875362319
{'C': 0.1. 'degree': 3. 'gamma': 0.1. 'kernel': 'rbf'}	0.91884058	0.91884058	0.91884058	0.91884058
{'C': 0.1. 'degree': 3. 'gamma': 1. 'kernel': 'linear'}	0.95942029	0.95942029	0.95942029	0.95942029
{'C': 0.1. 'degree': 3. 'gamma': 1. 'kernel': 'poly'}	0.971014493	0.971014493	0.971014493	0.971014493
{'C': 0.1. 'degree': 3. 'gamma': 1. 'kernel': 'rbf'}	0.939130435	0.939130435	0.939130435	0.939130435
{'C': 0.1. 'degree': 3. 'gamma': 'auto'. 'kernel': 'linear'}	0.95942029	0.95942029	0.95942029	0.95942029
{'C': 0.1. 'degree': 3. 'gamma': 'auto'. 'kernel': 'poly'}	0.336231884	0.336231884	0.336231884	0.336231884
{'C': 0.1. 'degree': 3. 'gamma': 'auto'. 'kernel': 'rbf'}	0.811594203	0.811594203	0.811594203	0.811594203
{'C': 0.1. 'degree': 4. 'gamma': 0.01. 'kernel': 'linear'}	0.95942029	0.95942029	0.95942029	0.95942029
{'C': 0.1. 'degree': 4. 'gamma': 0.01. 'kernel': 'poly'}	0.315942029	0.315942029	0.315942029	0.315942029
{'C': 0.1. 'degree': 4. 'gamma': 0.01. 'kernel': 'rbf'}	0.857971014	0.857971014	0.857971014	0.857971014
{'C': 0.1. 'degree': 4. 'gamma': 0.1. 'kernel': 'linear'}	0.95942029	0.95942029	0.95942029	0.95942029
{'C': 0.1. 'degree': 4. 'gamma': 0.1. 'kernel': 'poly'}	0.872463768	0.872463768	0.872463768	0.872463768
{'C': 0.1. 'degree': 4. 'gamma': 0.1. 'kernel': 'rbf'}	0.91884058	0.91884058	0.91884058	0.91884058
{'C': 0.1. 'degree': 4. 'gamma': 1. 'kernel': 'linear'}	0.95942029	0.95942029	0.95942029	0.95942029
{'C': 0.1. 'degree': 4. 'gamma': 1. 'kernel': 'poly'}	0.962318841	0.962318841	0.962318841	0.962318841
{'C': 0.1. 'degree': 4. 'gamma': 1. 'kernel': 'rbf'}	0.939130435	0.939130435	0.939130435	0.939130435
{'C': 0.1. 'degree': 4. 'gamma': 'auto'. 'kernel': 'linear'}	0.95942029	0.95942029	0.95942029	0.95942029
{'C': 0.1. 'degree': 4. 'gamma': 'auto'. 'kernel': 'poly'}	0.315942029	0.315942029	0.315942029	0.315942029
{'C': 0.1. 'degree': 4. 'gamma': 'auto'. 'kernel': 'rbf'}	0.811594203	0.811594203	0.811594203	0.811594203
{'C': 1. 'degree': 2. 'gamma': 0.01. 'kernel': 'linear'}	0.973913043	0.973913043	0.973913043	0.973913043
{'C': 1. 'degree': 2. 'gamma': 0.01. 'kernel': 'poly'}	0.805797101	0.805797101	0.805797101	0.805797101
{'C': 1. 'degree': 2. 'gamma': 0.01. 'kernel': 'rbf'}	0.942028986	0.942028986	0.942028986	0.942028986
{'C': 1. 'degree': 2. 'gamma': 0.1. 'kernel': 'linear'}	0.973913043	0.973913043	0.973913043	0.973913043
{'C': 1. 'degree': 2. 'gamma': 0.1. 'kernel': 'poly'}	0.953623188	0.953623188	0.953623188	0.953623188
{'C': 1. 'degree': 2. 'gamma': 0.1. 'kernel': 'rbf'}	0.982608696	0.982608696	0.982608696	0.982608696
{'C': 1. 'degree': 2. 'gamma': 1. 'kernel': 'linear'}	0.973913043	0.973913043	0.973913043	0.973913043
{'C': 1. 'degree': 2. 'gamma': 1. 'kernel': 'poly'}	0.973913043	0.973913043	0.973913043	0.973913043
{'C': 1. 'degree': 2. 'gamma': 1. 'kernel': 'rbf'}	0.968115942	0.968115942	0.968115942	0.968115942
{'C': 1. 'degree': 2. 'gamma': 'auto'. 'kernel': 'linear'}	0.973913043	0.973913043	0.973913043	0.973913043
{'C': 1. 'degree': 2. 'gamma': 'auto'. 'kernel': 'poly'}	0.666666667	0.666666667	0.666666667	0.666666667
{'C': 1. 'degree': 2. 'gamma': 'auto'. 'kernel': 'rbf'}	0.92173913	0.92173913	0.92173913	0.92173913
{'C': 1. 'degree': 3. 'gamma': 0.01. 'kernel': 'linear'}	0.973913043	0.973913043	0.973913043	0.973913043
{'C': 1. 'degree': 3. 'gamma': 0.01. 'kernel': 'poly'}	0.605797101	0.605797101	0.605797101	0.605797101
{'C': 1. 'degree': 3. 'gamma': 0.01. 'kernel': 'rbf'}	0.942028986	0.942028986	0.942028986	0.942028986
{'C': 1. 'degree': 3. 'gamma': 0.1. 'kernel': 'linear'}	0.973913043	0.973913043	0.973913043	0.973913043
{'C': 1. 'degree': 3. 'gamma': 0.1. 'kernel': 'poly'}	0.930434783	0.930434783	0.930434783	0.930434783
{'C': 1. 'degree': 3. 'gamma': 0.1. 'kernel': 'rbf'}	0.982608696	0.982608696	0.982608696	0.982608696
{'C': 1. 'degree': 3. 'gamma': 1. 'kernel': 'linear'}	0.973913043	0.973913043	0.973913043	0.973913043
{'C': 1. 'degree': 3. 'gamma': 1. 'kernel': 'poly'}	0.95942029	0.95942029	0.95942029	0.95942029

{'C': 1. 'degree': 3. 'gamma': 1. 'kernel': 'rbf'}	0.968115942	0.968115942	0.968115942	0.968115942
{'C': 1. 'degree': 3. 'gamma': 'auto'. 'kernel': 'linear'}	0.973913043	0.973913043	0.973913043	0.973913043
{'C': 1. 'degree': 3. 'gamma': 'auto'. 'kernel': 'poly'}	0.336231884	0.336231884	0.336231884	0.336231884
{'C': 1. 'degree': 3. 'gamma': 'auto'. 'kernel': 'rbf'}	0.92173913	0.92173913	0.92173913	0.92173913
{'C': 1. 'degree': 4. 'gamma': 0.01. 'kernel': 'linear'}	0.973913043	0.973913043	0.973913043	0.973913043
{'C': 1. 'degree': 4. 'gamma': 0.01. 'kernel': 'poly'}	0.4	0.4	0.4	0.4
{'C': 1. 'degree': 4. 'gamma': 0.01. 'kernel': 'rbf'}	0.942028986	0.942028986	0.942028986	0.942028986
{'C': 1. 'degree': 4. 'gamma': 0.1. 'kernel': 'linear'}	0.973913043	0.973913043	0.973913043	0.973913043
{'C': 1. 'degree': 4. 'gamma': 0.1. 'kernel': 'poly'}	0.92173913	0.92173913	0.92173913	0.92173913
{'C': 1. 'degree': 4. 'gamma': 0.1. 'kernel': 'rbf'}	0.982608696	0.982608696	0.982608696	0.982608696
{'C': 1. 'degree': 4. 'gamma': 1. 'kernel': 'linear'}	0.973913043	0.973913043	0.973913043	0.973913043
{'C': 1. 'degree': 4. 'gamma': 1. 'kernel': 'poly'}	0.968115942	0.968115942	0.968115942	0.968115942
{'C': 1. 'degree': 4. 'gamma': 1. 'kernel': 'rbf'}	0.968115942	0.968115942	0.968115942	0.968115942
{'C': 1. 'degree': 4. 'gamma': 'auto'. 'kernel': 'linear'}	0.973913043	0.973913043	0.973913043	0.973913043
{'C': 1. 'degree': 4. 'gamma': 'auto'. 'kernel': 'poly'}	0.315942029	0.315942029	0.315942029	0.315942029
{'C': 1. 'degree': 4. 'gamma': 'auto'. 'kernel': 'rbf'}	0.92173913	0.92173913	0.92173913	0.92173913
{'C': 10. 'degree': 2. 'gamma': 0.01. 'kernel': 'linear'}	0.979710145	0.979710145	0.979710145	0.979710145
{'C': 10. 'degree': 2. 'gamma': 0.01. 'kernel': 'poly'}	0.889855072	0.889855072	0.889855072	0.889855072
{'C': 10. 'degree': 2. 'gamma': 0.01. 'kernel': 'rbf'}	0.971014493	0.971014493	0.971014493	0.971014493
{'C': 10. 'degree': 2. 'gamma': 0.1. 'kernel': 'linear'}	0.979710145	0.979710145	0.979710145	0.979710145
{'C': 10. 'degree': 2. 'gamma': 0.1. 'kernel': 'poly'}	0.971014493	0.971014493	0.971014493	0.971014493
{'C': 10. 'degree': 2. 'gamma': 0.1. 'kernel': 'rbf'}	0.976811594	0.976811594	0.976811594	0.976811594
{'C': 10. 'degree': 2. 'gamma': 1. 'kernel': 'linear'}	0.979710145	0.979710145	0.979710145	0.979710145
{'C': 10. 'degree': 2. 'gamma': 1. 'kernel': 'poly'}	0.968115942	0.968115942	0.968115942	0.968115942
{'C': 10. 'degree': 2. 'gamma': 1. 'kernel': 'rbf'}	0.976811594	0.976811594	0.976811594	0.976811594
{'C': 10. 'degree': 2. 'gamma': 'auto'. 'kernel': 'linear'}	0.979710145	0.979710145	0.979710145	0.979710145
{'C': 10. 'degree': 2. 'gamma': 'auto'. 'kernel': 'poly'}	0.860869565	0.860869565	0.860869565	0.860869565
{'C': 10. 'degree': 2. 'gamma': 'auto'. 'kernel': 'rbf'}	0.965217391	0.965217391	0.965217391	0.965217391
{'C': 10. 'degree': 3. 'gamma': 0.01. 'kernel': 'linear'}	0.979710145	0.979710145	0.979710145	0.979710145
{'C': 10. 'degree': 3. 'gamma': 0.01. 'kernel': 'poly'}	0.814492754	0.814492754	0.814492754	0.814492754
{'C': 10. 'degree': 3. 'gamma': 0.01. 'kernel': 'rbf'}	0.971014493	0.971014493	0.971014493	0.971014493
{'C': 10. 'degree': 3. 'gamma': 0.1. 'kernel': 'linear'}	0.979710145	0.979710145	0.979710145	0.979710145
{'C': 10. 'degree': 3. 'gamma': 0.1. 'kernel': 'poly'}	0.962318841	0.962318841	0.962318841	0.962318841
{'C': 10. 'degree': 3. 'gamma': 0.1. 'kernel': 'rbf'}	0.976811594	0.976811594	0.976811594	0.976811594
{'C': 10. 'degree': 3. 'gamma': 1. 'kernel': 'linear'}	0.979710145	0.979710145	0.979710145	0.979710145
{'C': 10. 'degree': 3. 'gamma': 1. 'kernel': 'poly'}	0.968115942	0.968115942	0.968115942	0.968115942
{'C': 10. 'degree': 3. 'gamma': 1. 'kernel': 'rbf'}	0.976811594	0.976811594	0.976811594	0.976811594
{'C': 10. 'degree': 3. 'gamma': 'auto'. 'kernel': 'linear'}	0.979710145	0.979710145	0.979710145	0.979710145
{'C': 10. 'degree': 3. 'gamma': 'auto'. 'kernel': 'poly'}	0.663768116	0.663768116	0.663768116	0.663768116
{'C': 10. 'degree': 3. 'gamma': 'auto'. 'kernel': 'rbf'}	0.965217391	0.965217391	0.965217391	0.965217391
{'C': 10. 'degree': 4. 'gamma': 0.01. 'kernel': 'linear'}	0.979710145	0.979710145	0.979710145	0.979710145
{'C': 10. 'degree': 4. 'gamma': 0.01. 'kernel': 'poly'}	0.684057971	0.684057971	0.684057971	0.684057971
{'C': 10. 'degree': 4. 'gamma': 0.01. 'kernel': 'rbf'}	0.971014493	0.971014493	0.971014493	0.971014493
{'C': 10. 'degree': 4. 'gamma': 0.1. 'kernel': 'linear'}	0.979710145	0.979710145	0.979710145	0.979710145

{'C': 10. 'degree': 4. 'gamma': 0.1. 'kernel': 'poly'}	0.950724638	0.950724638	0.950724638	0.950724638
{'C': 10. 'degree': 4. 'gamma': 0.1. 'kernel': 'rbf'}	0.976811594	0.976811594	0.976811594	0.976811594
{'C': 10. 'degree': 4. 'gamma': 1. 'kernel': 'linear'}	0.979710145	0.979710145	0.979710145	0.979710145
{'C': 10. 'degree': 4. 'gamma': 1. 'kernel': 'poly'}	0.968115942	0.968115942	0.968115942	0.968115942
{'C': 10. 'degree': 4. 'gamma': 1. 'kernel': 'rbf'}	0.976811594	0.976811594	0.976811594	0.976811594
{'C': 10. 'degree': 4. 'gamma': 'auto'. 'kernel': 'linear'}	0.979710145	0.979710145	0.979710145	0.979710145
{'C': 10. 'degree': 4. 'gamma': 'auto'. 'kernel': 'poly'}	0.373913043	0.373913043	0.373913043	0.373913043
{'C': 10. 'degree': 4. 'gamma': 'auto'. 'kernel': 'rbf'}	0.965217391	0.965217391	0.965217391	0.965217391
{'C': 100. 'degree': 2. 'gamma': 0.01. 'kernel': 'linear'}	0.965217391	0.965217391	0.965217391	0.965217391
{'C': 100. 'degree': 2. 'gamma': 0.01. 'kernel': 'poly'}	0.953623188	0.953623188	0.953623188	0.953623188
{'C': 100. 'degree': 2. 'gamma': 0.01. 'kernel': 'rbf'}	0.982608696	0.982608696	0.982608696	0.982608696
{'C': 100. 'degree': 2. 'gamma': 0.1. 'kernel': 'linear'}	0.965217391	0.965217391	0.965217391	0.965217391
{'C': 100. 'degree': 2. 'gamma': 0.1. 'kernel': 'poly'}	0.973913043	0.973913043	0.973913043	0.973913043
{'C': 100. 'degree': 2. 'gamma': 0.1. 'kernel': 'rbf'}	0.979710145	0.979710145	0.979710145	0.979710145
{'C': 100. 'degree': 2. 'gamma': 1. 'kernel': 'linear'}	0.965217391	0.965217391	0.965217391	0.965217391
{'C': 100. 'degree': 2. 'gamma': 1. 'kernel': 'poly'}	0.968115942	0.968115942	0.968115942	0.968115942
{'C': 100. 'degree': 2. 'gamma': 1. 'kernel': 'rbf'}	0.976811594	0.976811594	0.976811594	0.976811594
{'C': 100. 'degree': 2. 'gamma': 'auto'. 'kernel': 'linear'}	0.965217391	0.965217391	0.965217391	0.965217391
{'C': 100. 'degree': 2. 'gamma': 'auto'. 'kernel': 'poly'}	0.915942029	0.915942029	0.915942029	0.915942029
{'C': 100. 'degree': 2. 'gamma': 'auto'. 'kernel': 'rbf'}	0.982608696	0.982608696	0.982608696	0.982608696
{'C': 100. 'degree': 3. 'gamma': 0.01. 'kernel': 'linear'}	0.965217391	0.965217391	0.965217391	0.965217391
{'C': 100. 'degree': 3. 'gamma': 0.01. 'kernel': 'poly'}	0.875362319	0.875362319	0.875362319	0.875362319
{'C': 100. 'degree': 3. 'gamma': 0.01. 'kernel': 'rbf'}	0.982608696	0.982608696	0.982608696	0.982608696
{'C': 100. 'degree': 3. 'gamma': 0.1. 'kernel': 'linear'}	0.965217391	0.965217391	0.965217391	0.965217391
{'C': 100. 'degree': 3. 'gamma': 0.1. 'kernel': 'poly'}	0.971014493	0.971014493	0.971014493	0.971014493
{'C': 100. 'degree': 3. 'gamma': 0.1. 'kernel': 'rbf'}	0.979710145	0.979710145	0.979710145	0.979710145
{'C': 100. 'degree': 3. 'gamma': 1. 'kernel': 'linear'}	0.965217391	0.965217391	0.965217391	0.965217391
{'C': 100. 'degree': 3. 'gamma': 1. 'kernel': 'poly'}	0.968115942	0.968115942	0.968115942	0.968115942
{'C': 100. 'degree': 3. 'gamma': 1. 'kernel': 'rbf'}	0.976811594	0.976811594	0.976811594	0.976811594
{'C': 100. 'degree': 3. 'gamma': 'auto'. 'kernel': 'linear'}	0.965217391	0.965217391	0.965217391	0.965217391
{'C': 100. 'degree': 3. 'gamma': 'auto'. 'kernel': 'poly'}	0.823188406	0.823188406	0.823188406	0.823188406
{'C': 100. 'degree': 3. 'gamma': 'auto'. 'kernel': 'rbf'}	0.982608696	0.982608696	0.982608696	0.982608696
{'C': 100. 'degree': 4. 'gamma': 0.01. 'kernel': 'linear'}	0.965217391	0.965217391	0.965217391	0.965217391
{'C': 100. 'degree': 4. 'gamma': 0.01. 'kernel': 'poly'}	0.814492754	0.814492754	0.814492754	0.814492754
{'C': 100. 'degree': 4. 'gamma': 0.01. 'kernel': 'rbf'}	0.982608696	0.982608696	0.982608696	0.982608696
{'C': 100. 'degree': 4. 'gamma': 0.1. 'kernel': 'linear'}	0.965217391	0.965217391	0.965217391	0.965217391
{'C': 100. 'degree': 4. 'gamma': 0.1. 'kernel': 'poly'}	0.962318841	0.962318841	0.962318841	0.962318841
{'C': 100. 'degree': 4. 'gamma': 0.1. 'kernel': 'rbf'}	0.979710145	0.979710145	0.979710145	0.979710145
{'C': 100. 'degree': 4. 'gamma': 1. 'kernel': 'linear'}	0.965217391	0.965217391	0.965217391	0.965217391
{'C': 100. 'degree': 4. 'gamma': 1. 'kernel': 'poly'}	0.968115942	0.968115942	0.968115942	0.968115942
{'C': 100. 'degree': 4. 'gamma': 1. 'kernel': 'rbf'}	0.976811594	0.976811594	0.976811594	0.976811594
{'C': 100. 'degree': 4. 'gamma': 'auto'. 'kernel': 'linear'}	0.965217391	0.965217391	0.965217391	0.965217391
{'C': 100. 'degree': 4. 'gamma': 'auto'. 'kernel': 'poly'}	0.666666667	0.666666667	0.666666667	0.666666667
{'C': 100. 'degree': 4. 'gamma': 'auto'. 'kernel': 'rbf'}	0.982608696	0.982608696	0.982608696	0.982608696

{'C': 1000. 'degree': 2. 'gamma': 0.01. 'kernel': 'linear'}	0.956521739	0.956521739	0.956521739	0.956521739
{'C': 1000. 'degree': 2. 'gamma': 0.01. 'kernel': 'poly'}	0.971014493	0.971014493	0.971014493	0.971014493
{'C': 1000. 'degree': 2. 'gamma': 0.01. 'kernel': 'rbf'}	0.982608696	0.982608696	0.982608696	0.982608696
{'C': 1000. 'degree': 2. 'gamma': 0.1. 'kernel': 'linear'}	0.956521739	0.956521739	0.956521739	0.956521739
{'C': 1000. 'degree': 2. 'gamma': 0.1. 'kernel': 'poly'}	0.968115942	0.968115942	0.968115942	0.968115942
{'C': 1000. 'degree': 2. 'gamma': 0.1. 'kernel': 'rbf'}	0.965217391	0.965217391	0.965217391	0.965217391
{'C': 1000. 'degree': 2. 'gamma': 1. 'kernel': 'linear'}	0.956521739	0.956521739	0.956521739	0.956521739
{'C': 1000. 'degree': 2. 'gamma': 1. 'kernel': 'poly'}	0.968115942	0.968115942	0.968115942	0.968115942
{'C': 1000. 'degree': 2. 'gamma': 1. 'kernel': 'rbf'}	0.976811594	0.976811594	0.976811594	0.976811594
{'C': 1000. 'degree': 2. 'gamma': 'auto'. 'kernel': 'linear'}	0.956521739	0.956521739	0.956521739	0.956521739
{'C': 1000. 'degree': 2. 'gamma': 'auto'. 'kernel': 'poly'}	0.968115942	0.968115942	0.968115942	0.968115942
{'C': 1000. 'degree': 2. 'gamma': 'auto'. 'kernel': 'rbf'}	0.976811594	0.976811594	0.976811594	0.976811594
{'C': 1000. 'degree': 3. 'gamma': 0.01. 'kernel': 'linear'}	0.956521739	0.956521739	0.956521739	0.956521739
{'C': 1000. 'degree': 3. 'gamma': 0.01. 'kernel': 'poly'}	0.930434783	0.930434783	0.930434783	0.930434783
{'C': 1000. 'degree': 3. 'gamma': 0.01. 'kernel': 'rbf'}	0.982608696	0.982608696	0.982608696	0.982608696
{'C': 1000. 'degree': 3. 'gamma': 0.1. 'kernel': 'linear'}	0.956521739	0.956521739	0.956521739	0.956521739
{'C': 1000. 'degree': 3. 'gamma': 0.1. 'kernel': 'poly'}	0.95942029	0.95942029	0.95942029	0.95942029
{'C': 1000. 'degree': 3. 'gamma': 0.1. 'kernel': 'rbf'}	0.965217391	0.965217391	0.965217391	0.965217391
{'C': 1000. 'degree': 3. 'gamma': 1. 'kernel': 'linear'}	0.956521739	0.956521739	0.956521739	0.956521739
{'C': 1000. 'degree': 3. 'gamma': 1. 'kernel': 'poly'}	0.968115942	0.968115942	0.968115942	0.968115942
{'C': 1000. 'degree': 3. 'gamma': 1. 'kernel': 'rbf'}	0.976811594	0.976811594	0.976811594	0.976811594
{'C': 1000. 'degree': 3. 'gamma': 'auto'. 'kernel': 'linear'}	0.956521739	0.956521739	0.956521739	0.956521739
{'C': 1000. 'degree': 3. 'gamma': 'auto'. 'kernel': 'poly'}	0.88115942	0.88115942	0.88115942	0.88115942
{'C': 1000. 'degree': 3. 'gamma': 'auto'. 'kernel': 'rbf'}	0.976811594	0.976811594	0.976811594	0.976811594
{'C': 1000. 'degree': 4. 'gamma': 0.01. 'kernel': 'linear'}	0.956521739	0.956521739	0.956521739	0.956521739
{'C': 1000. 'degree': 4. 'gamma': 0.01. 'kernel': 'poly'}	0.872463768	0.872463768	0.872463768	0.872463768
{'C': 1000. 'degree': 4. 'gamma': 0.01. 'kernel': 'rbf'}	0.982608696	0.982608696	0.982608696	0.982608696
{'C': 1000. 'degree': 4. 'gamma': 0.1. 'kernel': 'linear'}	0.956521739	0.956521739	0.956521739	0.956521739
{'C': 1000. 'degree': 4. 'gamma': 0.1. 'kernel': 'poly'}	0.962318841	0.962318841	0.962318841	0.962318841
{'C': 1000. 'degree': 4. 'gamma': 0.1. 'kernel': 'rbf'}	0.965217391	0.965217391	0.965217391	0.965217391
{'C': 1000. 'degree': 4. 'gamma': 1. 'kernel': 'linear'}	0.956521739	0.956521739	0.956521739	0.956521739
{'C': 1000. 'degree': 4. 'gamma': 1. 'kernel': 'poly'}	0.968115942	0.968115942	0.968115942	0.968115942
{'C': 1000. 'degree': 4. 'gamma': 1. 'kernel': 'rbf'}	0.976811594	0.976811594	0.976811594	0.976811594
{'C': 1000. 'degree': 4. 'gamma': 'auto'. 'kernel': 'linear'}	0.956521739	0.956521739	0.956521739	0.956521739
{'C': 1000. 'degree': 4. 'gamma': 'auto'. 'kernel': 'poly'}	0.8	0.8	0.8	0.8
{'C': 1000. 'degree': 4. 'gamma': 'auto'. 'kernel': 'rbf'}	0.976811594	0.976811594	0.976811594	0.976811594
